# Supplementary material for: A late-stage assembly checkpoint of the human mitochondrial ribosome large subunit
Source: Nat Commun. 2022 Feb 17;13:929. doi: 10.1038/s41467-022-28503-5 (PMC8854578; doi:10.1038/s41467-022-28503-5)
Supplement: Supplementary file 1 — Supplementary Information [file 41467_2022_28503_MOESM1_ESM.pdf]

SUPPLEMENTARY INFORMATION FOR:

A late-stage assembly checkpoint of the human mitochondrial ribosome large subunit

Pedro Rebelo-Guiomar[1], Simone Pellegrino[2,3,4], Kyle C. Dent[2,3,4,8], Aldema Sas-Chen[5,8], Leonor Miller-Fleming[1], Caterina Garone[1,8], Lindsey Van Haute[1], Jack F. Rogan[6], Adam Dinan[7], Andrew E. Firth[7], Byron Andrews[6], Alexander J. Whitworth[1], Schraga Schwartz[5], Alan J. Warren[2,3,4], Michal Minczuk[1,9]

[1] MRC Mitochondrial Biology Unit, University of Cambridge, Cambridge Biomedical Campus, Keith Peters Building, Hills Rd, Cambridge CB2 0XY, United Kingdom.

[2] Cambridge Institute for Medical Research, University of Cambridge, Cambridge Biomedical Campus, Keith Peters Building, Hills Rd, Cambridge CB2 0XY, United Kingdom.

[3] Wellcome Trust – MRC Stem Cell Institute, Cambridge Biomedical Campus, Jeffrey Cheah Biomedical Centre, Puddicombe Way, Cambridge, CB2 0AW, United Kingdom.

[4] Department of Haematology, School of Clinical Medicine, University of Cambridge, Cambridge Biomedical Campus, Jeffrey Cheah Biomedical Centre, Puddicombe Way, Cambridge, CB2 0AW, United Kingdom.

[5] Department of Molecular Genetics, Weizmann Institute of Science, Rehovot 76100, Israel.

[6] STORM Therapeutics Limited, Babraham Research Campus, Moneta Building, Cambridge, CB22 3AT, United Kingdom.

[7] Department of Pathology, University of Cambridge, Tennis Court Road, Cambridge, CB2 1QP, United Kingdom.

[8] Current address – K.C.D.: MRC Laboratory of Molecular Biology, Cambridge Biomedical Campus, Francis Crick Avenue, Cambridge CB2 0QH, United Kingdom. A.S.-C.: Shmunis School of Biomedicine and Cancer Research, The George S. Wise Faculty of Life Sciences, Tel Aviv University, Tel Aviv 6997801, Israel. C.G.: Department of Medical and Surgical Sciences, University of Bologna, Bologna 40126, Italy.

[9] Correspondence: michal.minczuk@mrc-mbu.cam.ac.uk (M.M.)

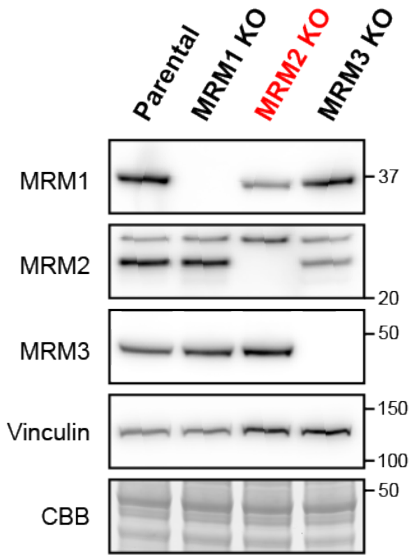

Supplementary Fig. 1 | Validation of the MRM1, MRM2, and MRM3 knock-out cell lines. Immunodetection of the three mitochondrial 2'-*O*-methyltransferases in cellular lysates from parental and each of the knock-out cell lines. Molecular weights of protein standards are presented in kDa to the right of each blot. Coomassie brilliant blue (CBB) staining is shown as a loading indicator. This experiment was replicated twice with similar results.

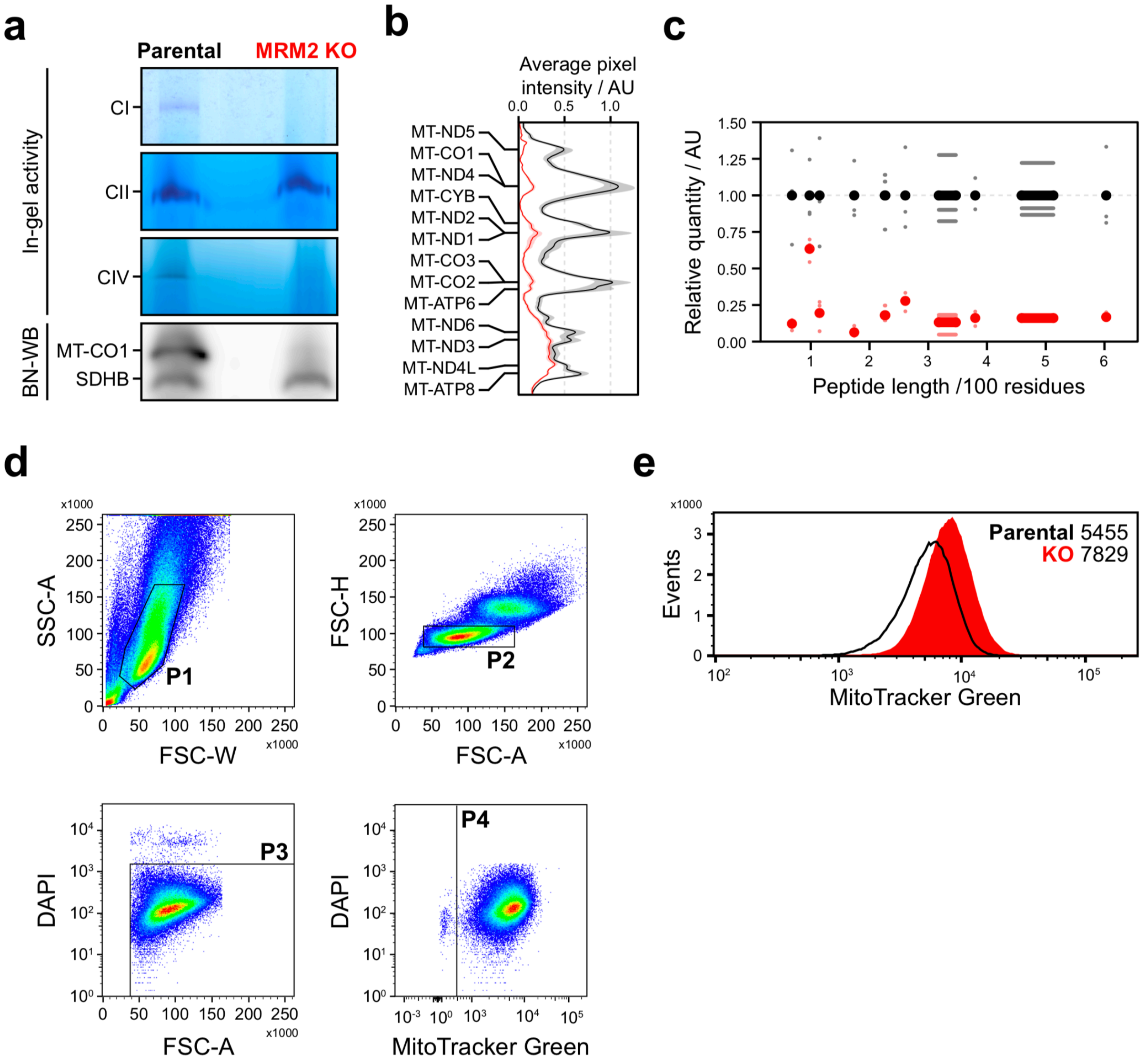

Supplementary Fig. 2 | Investigation of synthesis, assembly and activity of OxPhos complexes in the absence of MRM2. a, NADH oxidase (CI), succinate oxidase (CII) and cytochrome *c* oxidase (CIV) activities evaluated in cell extracts. Steady-state levels of complexes IV (MT-CO1) and II (SDHB) were evaluated by immunoblotting of cellular contents separated in near-native conditions. This experiment was replicated twice with similar results. **b**, Average profile of mtDNA-encoded proteins observed in Fig. 3b (n = 3). Traces from parental and MRM2-depleted samples are presented in black and red, respectively. **c**, Quantification of mtDNA-encoded proteins from metabolic labelling (Fig. 3b) plotted against their peptide length. Datapoints (small dots) and corresponding mean values (n = 3, larger solid dots) representing samples from parental and MRM2-depleted cells are presented in black and red, respectively. **d**, Flow cytometric assessment of mitochondrial mass (P1: cells; P2: singlets; P3: viable cells; P4: cells stained with

MitoTracker Green). All plot axes represent fluorescence intensity in arbitrary fluorescence units. **e**, MitoTracker Green fluorescence intensity was assessed in events gated in P4 (shown in the top right corner for each cell line), and the resulting histogram is shown. Source data are provided as a Source Data file.

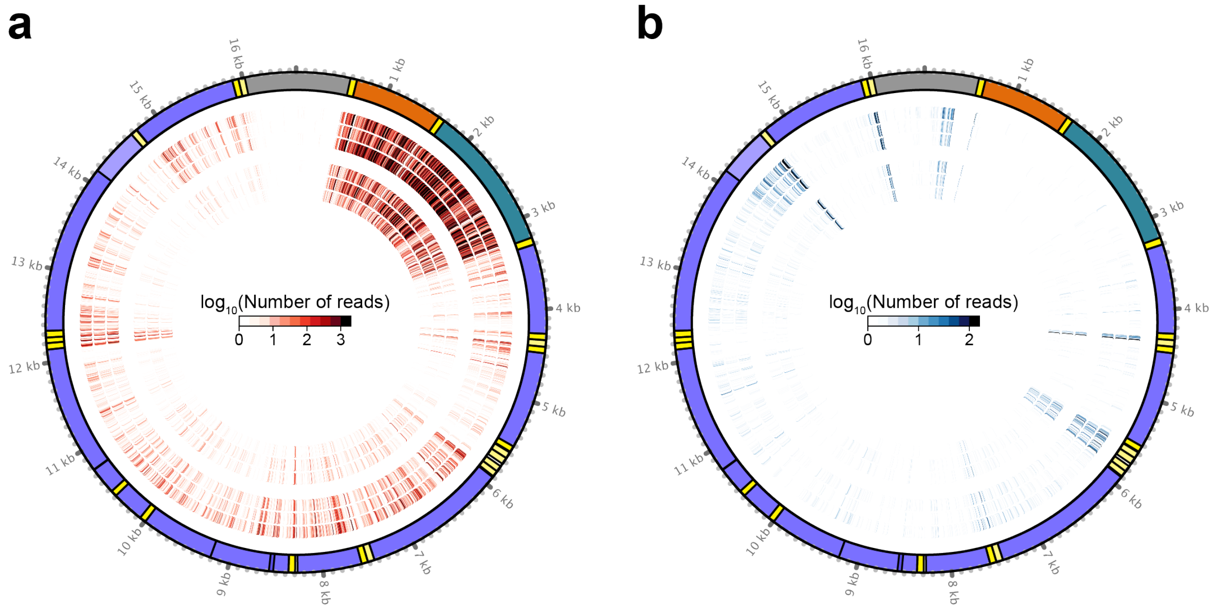

Supplementary Fig. 3 | Distribution of ribosomal occupancy along mitochondrial transcripts. Overview of the mitochondrial ribosome footprints mapped to the mitochondrial reference sequence. **a**, Heavy-strand promoter- and **b**, light-strand promoter- derived reads. The 5' read ends from the triplicate parental samples are presented in the three outer rings and those from MRM2 knock-out samples are presented in the three inner rings. The mitochondrial transcriptome is represented in a circular plot to facilitate visual comprehension. Yellow: mt-tRNAs, purple: mt-mRNAs, orange: 12S mt-rRNA, teal: 16S mt-rRNA, grey: D-loop. Transcripts encoded in the anti-sense strand are represented in the circular ideogram as lighter crown arcs. Mitoribosome occupancy is presented for each mitochondrial transcript in Fig. 3c.

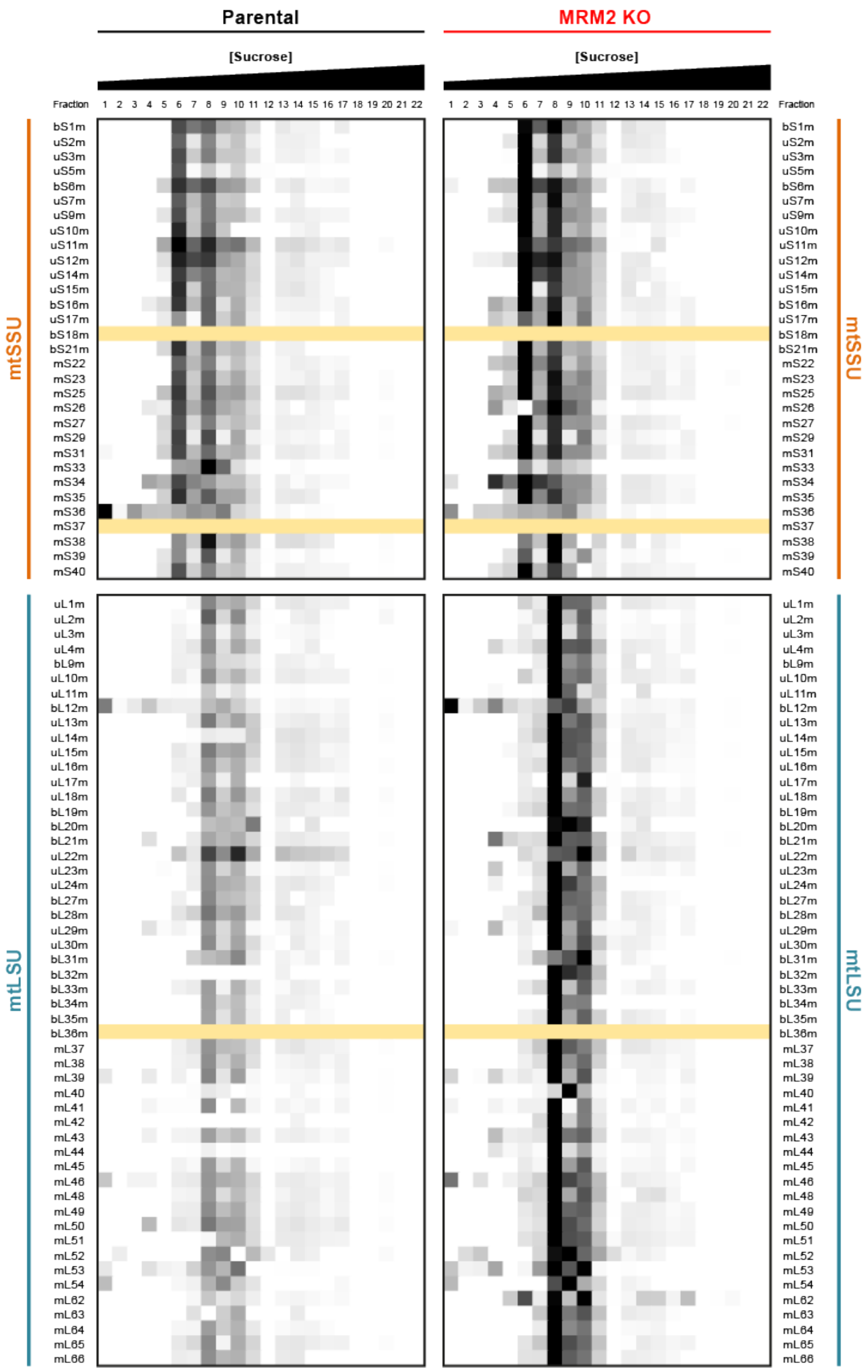

Supplementary Fig. 4 | Proteomic characterisation of mitoribosome components. Heatmap of the quantitative profile of mitoribosomal proteins in fractions collected from a continuous sucrose density gradient where mitochondrial extracts were resolved. The profile of each detected protein is coloured according to their abundance in each fraction (low to high abundance is coloured from white to black, respectively). Proteins for which no peptides were detected in parental and *MRM2* knock-out samples are shown in yellow. A summary of these results is presented in Fig. 4.

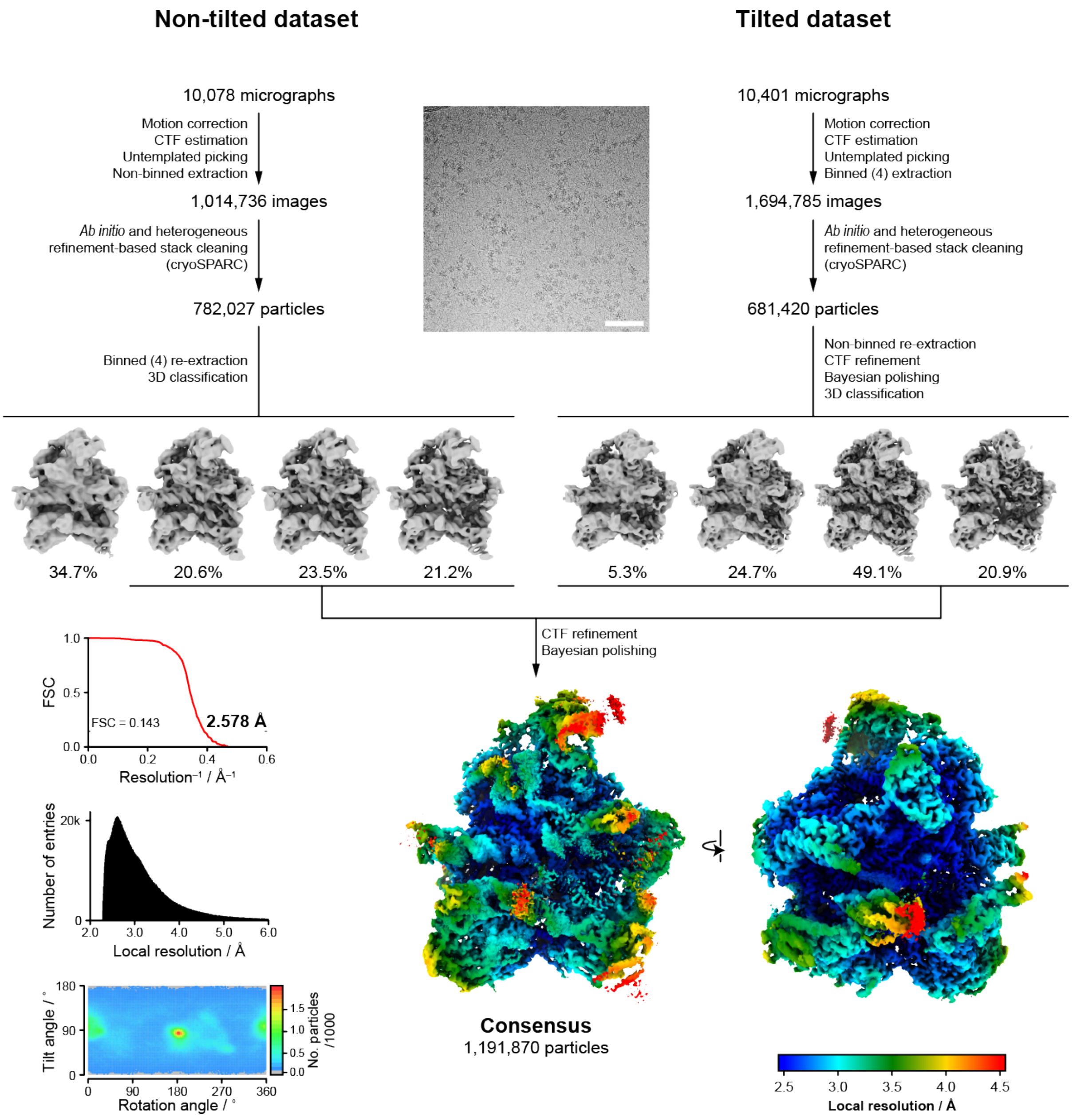

Supplementary Fig. 5 | CryoEM data processing workflow. Processing strategy is presented for the collected datasets when worked separately, and after being merged. An example micrograph is shown (scale bar: 100 nm), as well as the outcome maps of key classification steps (particle distribution is presented as percentages below the map of each class) and final consensus maps (coloured by local resolution). Fourier shell correlation (FSC), local resolution distribution and angular distribution are presented in their respective plots. Related to Fig. 5a.

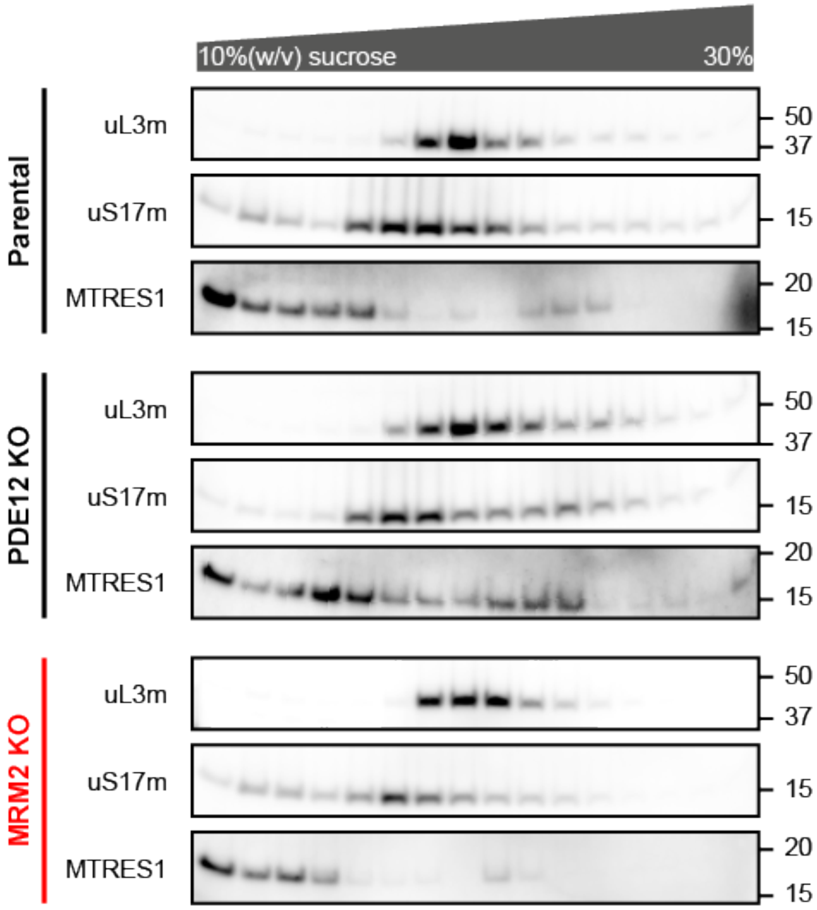

Supplementary Fig. 6 | Investigation of the presence of mtLSU-associated recycling factors. Immunoblot profile of mtLSU (uL3m), mtSSU (uS17m) and the recycling factor MTRES1 along a continuous density gradient. Molecular weights of protein standards are presented in kDa to the right of each blot. This experiment was replicated twice with similar results.

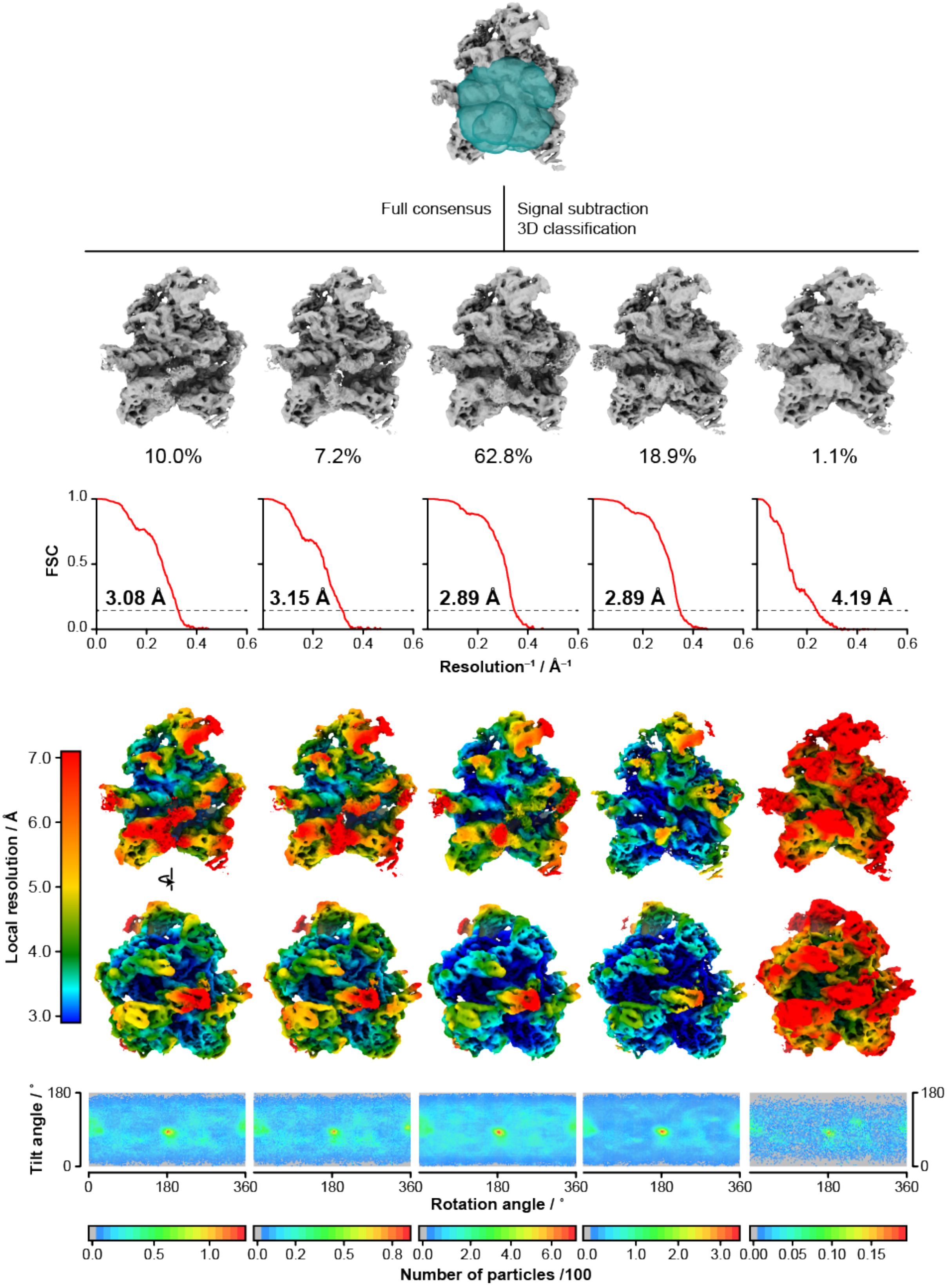

Supplementary Fig. 7 | Details of the focused classification on the intersubunit interface. A mask containing the intersubunit interface (teal surface) was used to perform signal subtraction. Classification (T=300) of the resulting signal generates the five presented classes (state 1 to 5 from left to right; particle distribution is presented

as percentages below the map of each class). Fourier shell correlation (FSC), surface colouring by local resolution (top: intersubunit interface view; bottom: 180° turn, peptide exit tunnel view), and angular distribution are presented. Overall resolution of each map is presented for FSC=0.143 (dashed line). Related to Fig. 5b.

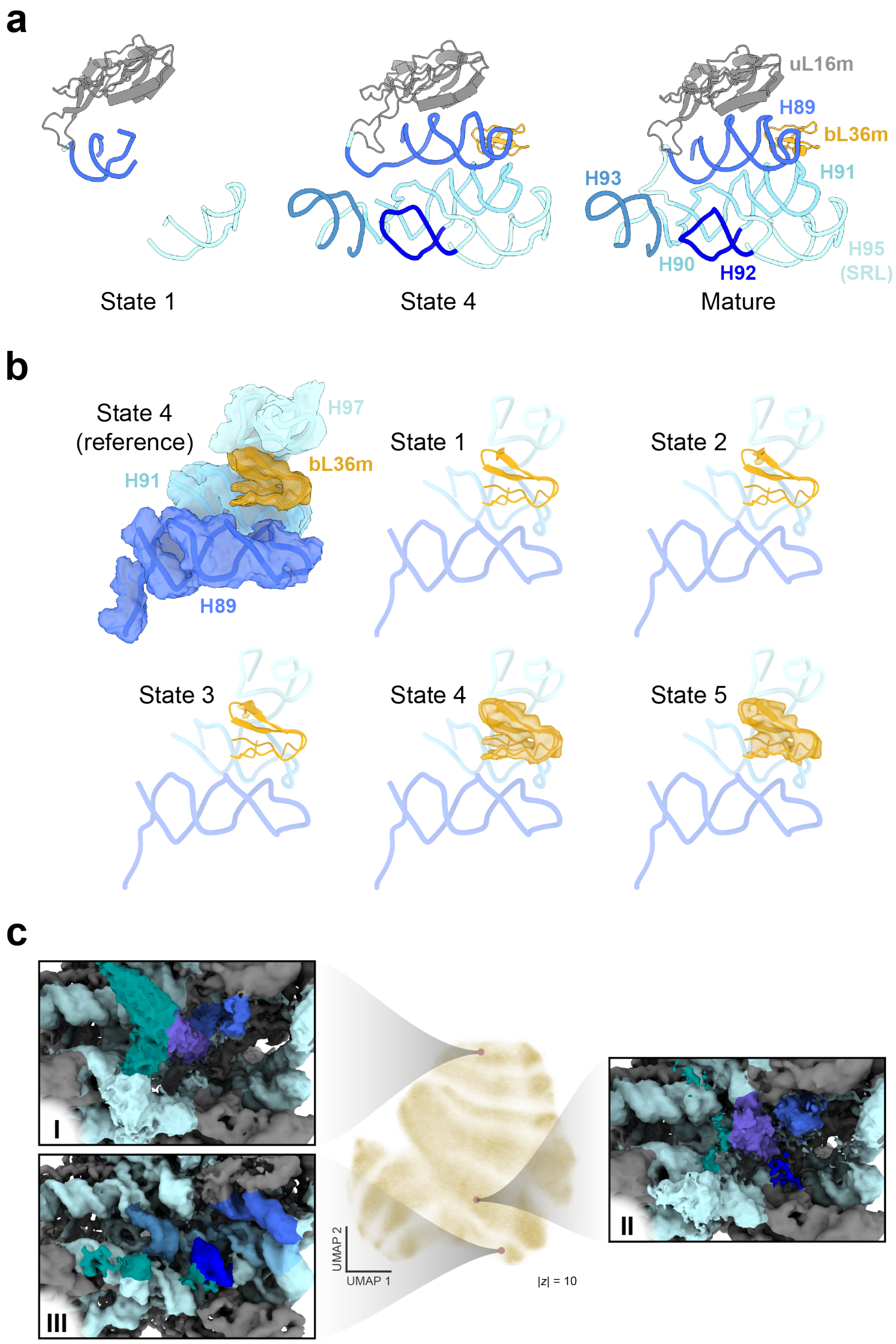

a
uL16m
H89
bL36m
H91
H93
H95
(SRL)
H90
H92
State 1
State 4
Mature
b
State 4
(reference)
H97
bL36m
H91
H89
State 1
State 2
State 3
State 4
State 5
c
I
II
III
UMAP 2
UMAP 1
|z| = 10

Supplementary Fig. 8 | Inspection of heterogeneity of mtLSU intersubunit interface components. a, Conformational and compositional heterogeneity of H89-93, H95 (containing the sarcin–ricin loop, SRL) and associated ribosomal proteins. Comparison of the modelled assembly intermediate states with the structure of the mature mtLSU (PDB 3J9M). **b**, Evaluation of bL36m density (dark orange surface) across the different mtLSU conformation states. The model of state 4 is shown for all states merely as a guide for the expected (states 1-3) or actual (states 4 and 5) location of H89 and bL36m. H91 (domain V) and H97 (domain IV) are shown as proximal interactors of bL36m in the L7/L12 stalk. **c**, Recapitulation of structural intermediates using a neural network approach (cryoDRGN). Uniform manifold approximation and projection (UMAP) representation of the latent space is shown (centre), alongside highlighted representations of selected cluster centres (left and right). Latent space exploration is shown in greater extension in Supplementary Movie 1. Surfaces and ribbons are presented with the same colour code as Fig. 5a.

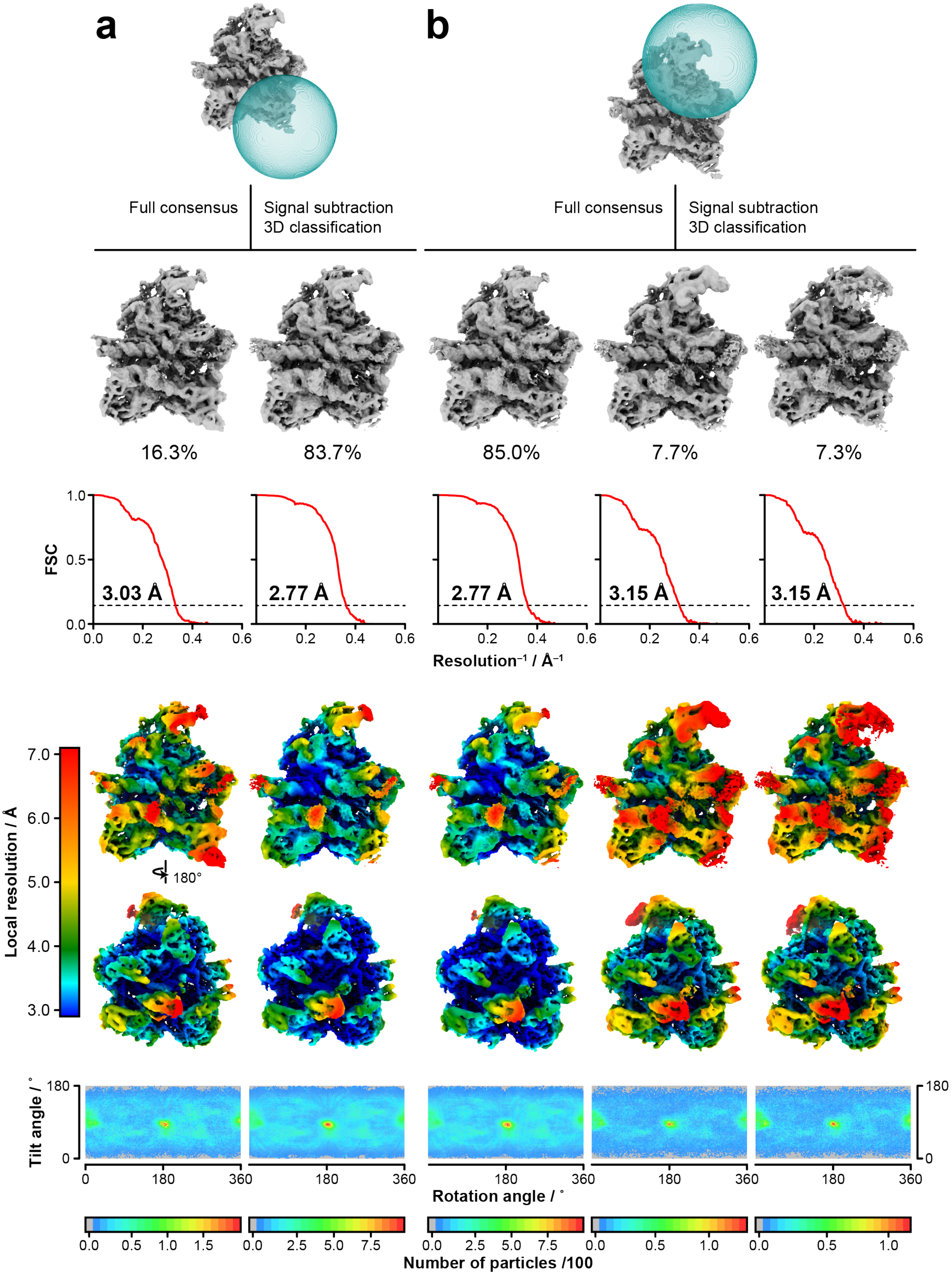

Supplementary Fig. 9 | Details of the focused classification on additional mtLSU regions. Masks (teal surface) containing **a**, the MALSU1:L0R8F8:mtACP module or **b**, the central protuberance were used to perform signal subtraction. Classification (T=300 for MALSU1 module, T=50 for central protuberance) of the resulting signal

generates the presented classes (particle distribution is presented as percentages below the map of each class). Fourier shell correlation (FSC), surface colouring by local resolution (top: intersubunit interface view; bottom: 180° turn, peptide exit tunnel view), and angular distribution are presented. Overall resolution of each map is presented for FSC=0.143 (dashed line).

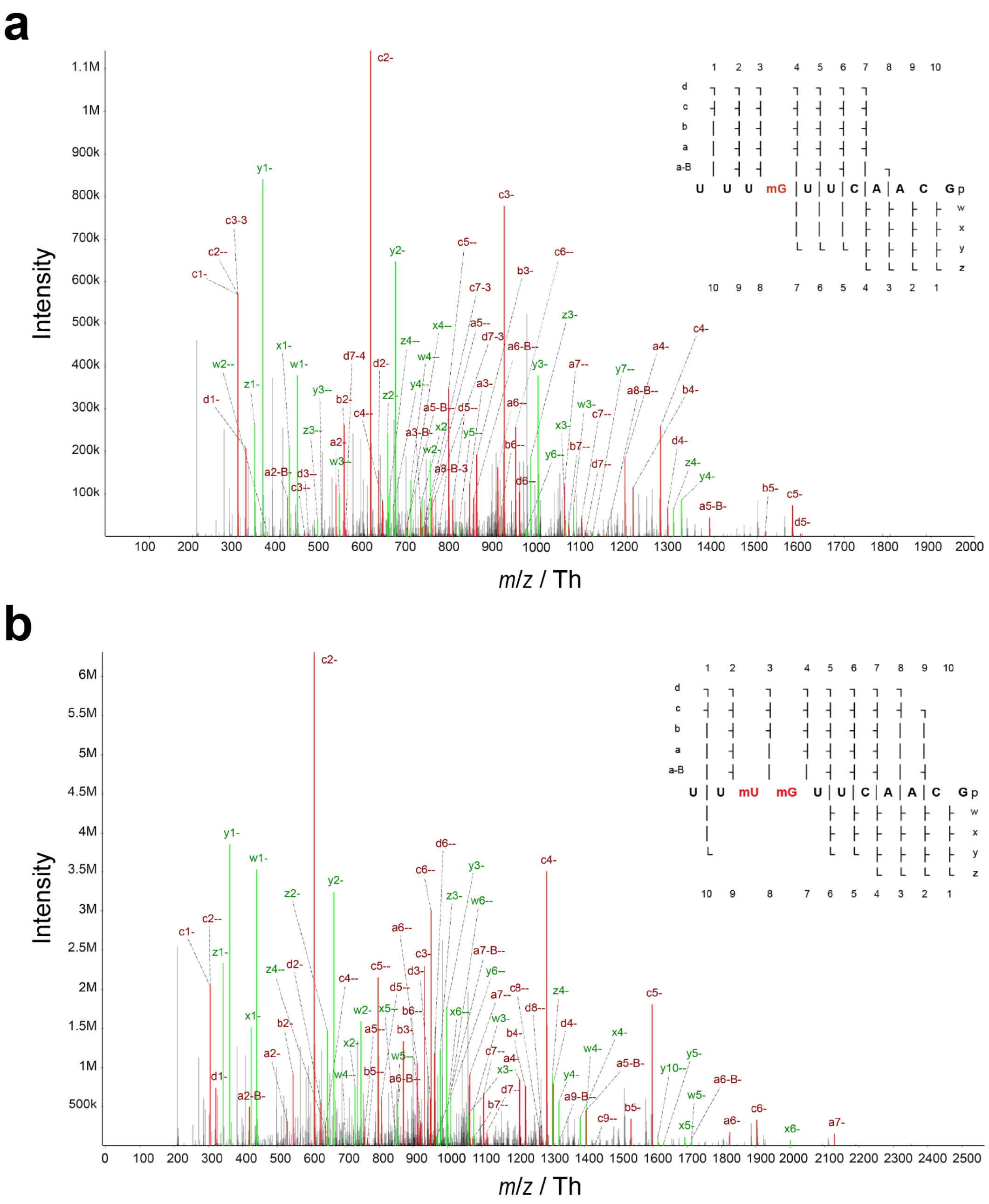

Supplementary Fig. 10 | Mass spectrometric characterisation of 16S mt-rRNA oligonucleotides. Annotated RNA MS2 fragmentation spectra from RNase T1 cleavage products of **a**, U3039/Gm3040 (maximum ion score: 198.87; Q value: 0) and **b**, Um3039/Gm3040 (maximum ion score: 220.06; Q value: 0) modified 16S mt-rRNA. Scores were generated during assignment by NucleicAcidSearchEngine. Related to Fig. 6b.

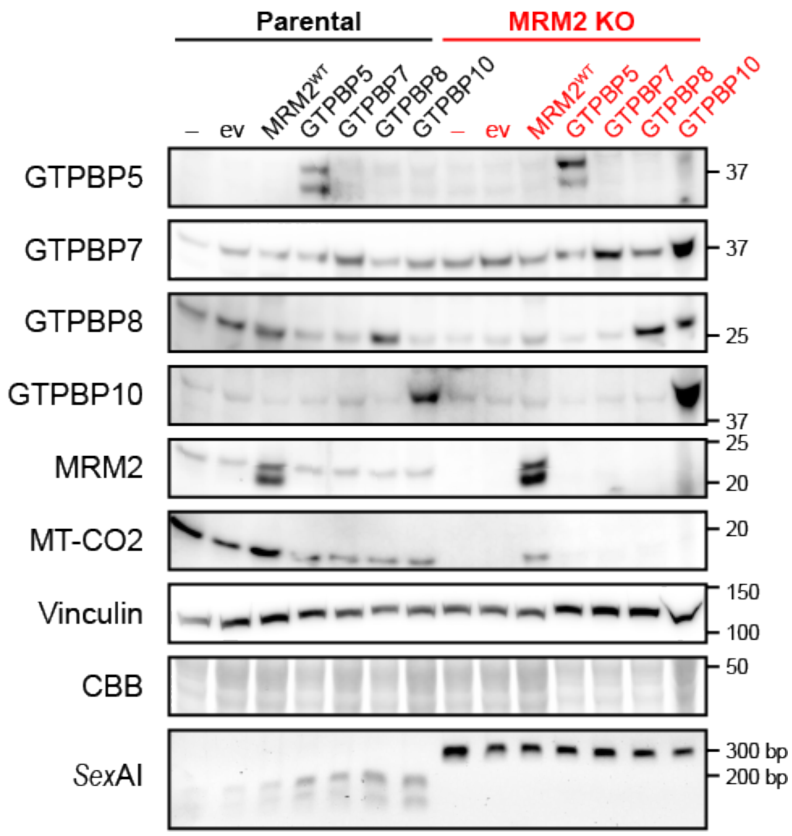

Supplementary Fig. 11 | Investigation on the functional rescue of mitochondrial translation in MRM2-depleted cells by mitochondrial GTPBPs. Immunoblot evaluation of functional rescue of mitochondrial translation in cell lines complemented with wild-type MRM2 (MRM2^{WT}), as well as GTPBP5/MTG2, GTPBP7/MTG1, GTPBP8 and GTPBP10. Molecular weights of protein standards are presented in kDa to the right of each blot. Coomassie brilliant blue (CBB) staining is shown as a loading indicator. For each cell line, electrophoretically separated *Sex*AI-digested amplicons of the genomic *MRM2* locus targeted for gene editing are presented. This experiment was replicated twice with similar results.

Supplementary Table 1 | CryoEM data collection parameters.

	Non-tilted data collection	Tilted data collection
**Data collection and processing**		
Microscope	Titan Krios	Titan Krios
Detector	Falcon 3EC	Falcon 3EC
Magnification	75,000x	75,000x
Voltage (kV)	300	300
Electron exposure (e^{-} Å^{-2})	52.5	52.1
Defocus range (μm)	−2.6 to −1.0	−2.2 to −1.4
Pixel size (Å)	1.06	1.06
Stage tilt (°)	0	−20
Initial particle images (no.)	1,014,736	1,694,785
Final particle images (no.)	510,450	681,420

Supplementary Table 2 | Materials and resources.

MATERIAL / RESOURCE	SOURCE	IDENTIFIER
Mouse monoclonal anti- β-actin	Sigma-Aldrich	A2228
Rabbit polyclonal anti-GTPBP5	Atlas Antibodies	HPA047379
Rabbit polyclonal anti-GTPBP7	Atlas Antibodies	HPA037827
Rabbit polyclonal anti-GTPBP8	Atlas Antibodies	HPA034831
Rabbit polyclonal anti-GTPBP10	Atlas Antibodies	HPA021076
Rabbit polyclonal anti-MRM1	Atlas Antibodies	HPA021598
Mouse monoclonal anti-MRM2	MyBioSource	MBS120390
Rabbit polyclonal anti-MRM3	Atlas Antibodies	HPA022534
Mouse monoclonal anti-MT-CO1	Abcam	ab14705
Mouse monoclonal anti-MT-CO2	Abcam	ab110258
Rabbit polyclonal anti-MTRES1	Atlas Antibodies	HPA049535
Mouse monoclonal anti-SDHB	Abcam	ab14714
Mouse monoclonal anti-TOM22	Abcam	ab10436
Rabbit polyclonal anti-uL3m	Proteintech	16584-1-AP
Rabbit polyclonal anti-uS17m	Proteintech	18881-1-AP
Mouse monoclonal anti-Vinculin	Sigma-Aldrich	V4505
Rabbit polyclonal anti-COX4	Kindly provided by Edward Owusu-Ansah	(Murari *et al.*, 2020)
Rabbit polyclonal anti-mt:ND1	Kindly provided by Edward Owusu-Ansah	(Murari *et al.*, 2020)
Mouse monoclonal anti-PDHA1	Abcam	ab110334
Rabbit polyclonal anti-SDHA	Kindly provided by Edward Owusu-Ansah	(Murari *et al.*, 2020)
Rabbit polyclonal anti-UQCR-C2	Kindly provided by Edward Owusu-Ansah	(Murari *et al.*, 2020)
Goat anti-mouse IgG (H+L), HRP Conjugate	Promega	W4021
Goat anti-rabbit IgG (H+L), HRP Conjugate	Promega	W4011
Acetonitrile	Romil	H049
Acid-Phenol:Chloroform, pH 4.5, with IAA, 125:24:1	Invitrogen	AM9720
Ammonium bicarbonate	Fluka	09830
Antimycin A	Sigma-Aldrich	A8674
L-arginine	Sigma-Aldrich	A8094
$^{13}C_6$,$^{15}N_4$-L-arginine	Sigma-Aldrich	608033
BAM15	Tim Tec	ST056388
Benzonase	Merk Millipore	70746
Bovine Serum Albumin (BSA)	VWR	A8674
Catalase	Sigma-Aldrich	C9322
Chloramphenicol	Alfa Aesar	B20841
cOmplete EDTA-free Protease Inhibitor Cocktail	Roche	11873580001
cOmplete Protease Inhibitor Cocktail	Roche	11697498001
Cycloheximide	TOKU-E	C001
Cytochrome c	Sigma-Aldrich	C7752
L-cystine dihydrochloride	Alfa Aesar	J62292
3,3'-diaminobenzidine tetrahydrochloride hydrate	Sigma-Aldrich	D5637
Dialysed Fetal Bovine Serum	Sigma-Aldrich	F0392
Dithiothreitol (DTT)	Melford	MB1015
DMEM for SILAC	Thermo Scientific	A33822
DMEM, high glucose, GlutaMAX Supplement, pyruvate	Gibco	31966047
DMEM, high glucose, no methionine, no cystine	Gibco	21013024

DMEM, high glucose, no sodium bicarbonate, powder	Sigma-Aldrich	D7777
DMEM, no glucose	Gibco	11966025
n-dodecyl-β-D-maltoside (DDM)	Sigma-Aldrich	D4641
Emetine	Sigma-Aldrich	E2375
Ethanol	Sigma-Aldrich	32221
Fetal Bovine Serum	Gibco	10270106
Formic acid	VWR	450122M
FuGENE 6 Transfection Reagent	Promega	E2691
Galactose	Sigma-Aldrich	G5388
Glucose	Sigma-Aldrich	G8270
GlutaMAX	Gibco	35050061
Hexafluoroisopropanol (HFIP)	Apollo Scientific	N/A
L-lysine	Sigma-Aldrich	L8662
$^{13}C_6$,$^{15}N_2$-L-lysine	Cambridge Isotope Laboratories	CNLM-291-H-0.5
Magnesium chloride	Fisher Scientific	BP214
Mannitol	Sigma-Aldrich	M9546
Methanol	Fisher Scientific	A456-212
[^{35}S]-L-methionine	Perkin Elmer	NEG009L005MC
NADH	Roche	10107735001
Nitro blue tetrazolium	Sigma-Aldrich	N5514
Oligomycin	Sigma-Aldrich	O4876
L-proline	Sigma-Aldrich	P5607
Penicillin-Streptomycin	Gibco	15070063
Phenazine methosulfate	Sigma-Aldrich	P9625
Phenylmethylsulfonyl fluoride (PMSF)	Sigma-Aldrich	P7626
Poly-L-lysine	Sigma-Aldrich	P6282
Potassium chloride	Fisher Scientific	P/4280/53
Proteinase K	New England Biolabs	P8107S
Puromycin	InvivoGen	ant-pr
RNAse I	Thermo Scientific	EN0601
Rotenone	Sigma-Aldrich	R8875
Coomassie Brilliant Blue (SimplyBlue Safestain)	Invitrogen	LC6065
Sodium acetate	Fisher Scientific	S/2120/53
Sodium bicarbonate	Sigma-Aldrich	S5761
Sodium carbonate	VWR	102404H
Sodium chloride	Sigma-Aldrich	S3014
Sodium pyruvate	Gibco	11360070
Sodium succinate	Sigma-Aldrich	S2378
Sucrose	Sigma-Aldrich	S9378
SUPERase•In RNase Inhibitor	Invitrogen	AM2694
TRI Reagent	Sigma-Aldrich	T9424
Triethylamine (TEA)	VWR	N/A
Trypsin (sequencing grade)	Roche	11418475001
Triton X-100	Fluka	93420
TRIzol LS Reagent	Invitrogen	10296028
TRIzol reagent	Invitrogen	15596026
Uridine	Sigma-Aldrich	U3750
Water (UHPLC grade)	Fisher Scientific	W/0120/PB15
Bolt 4-12% Bis-Tris Plus gels	Invitrogen	NW04120BOX
Cell Line Nucleofector Kit V	Lonza	VCA-1003

CompoZr Knockout Zinc Finger Nucleases: FTSJ2	Sigma-Aldrich	CKOZFND8984-1KT
CyQUANT Cell Proliferation Assay Kit	Thermo Fisher Scientific	C7026
ECL Prime Western Blotting Detection Reagent	Amersham	RPN2232
FastAP Thermosensitive Alkaline Phosphatase	Thermo Scientific	EF0651
Maxima H Minus cDNA Synthesis Master Mix	Thermo Scientific	M1661
PowerUp SYBR Green Master Mix	Applied Biosystems	A25741
Ribo-Zero Gold Kit	Illumina	MRZG126
RNA ScreenTape	Agilent	5067-5576
RNAse T1	Sigma-Aldrich	N/A
Seahorse XFe96 Extracellular Flux Assay Kit	Agilent	102601-100
SexAI	New England Biolabs	R0605S
SuperScript III Reverse Transcriptase	Invitrogen	18080093
T4 DNA ligase	New England Biolabs	M0202S
T4 RNA ligase 1	New England Biolabs	M0204S
TruSeq Small RNA Library Preparation Kit	Illumina	RS-200-0012
TURBO DNA-free Kit	Invitrogen	AM1907
Turbo DNase	Invitrogen	AM2238
Human reference genome NCBI build 38, GRCh38.p13	Genome Reference Consortium	http://www.ncbi.nlm.nih.gov/projects/genome/assembly/grc/human/
Human mitochondrial revised Cambridge Reference Sequence, rCRS	GenBank	NCBI NC_012920.1
Transcriptome-wide analysis of 2'-*O*-methylation in human mitochondria	This paper	GEO: GSE179085
Mitochondrial ribosome footprinting	This paper	ArrayExpress E-MTAB-11292
Structure of a native assembly intermediate of the human mitochondrial ribosome with unfolded interfacial rRNA	(Brown *et al.*, 2017)	PDB: 5OOL
Intersubunit interface FCwSS, state 1 cryoEM map	This paper	EMDB EMD-13965
Intersubunit interface FCwSS, state 2 cryoEM map	This paper	EMDB EMD-13962
Intersubunit interface FCwSS, state 3 cryoEM map	This paper	EMDB EMD-13963
Intersubunit interface FCwSS, state 4 cryoEM map	This paper	EMDB EMD-13967
Intersubunit interface FCwSS, state 5 cryoEM map	This paper	EMDB EMD-13966
Intersubunit interface FCwSS, state 1 atomic coordinates	This paper	PDB 7QH6
Intersubunit interface FCwSS, state 4 atomic coordinates	This paper	PDB 7QH7
HEK 293T Flp-In T-REx	Invitrogen	R78007
HEK 293T Flp-In T-REx + empty vector	This paper	N/A
HEK 293T Flp-In T-REx + MRM2 WT	This paper	N/A
HEK 293T Flp-In T-REx + MRM2 K59A	This paper	N/A
HEK 293T Flp-In T-REx + MRM2 D154A	This paper	N/A
HEK 293T Flp-In T-REx + GTPBP5	This paper	N/A
HEK 293T Flp-In T-REx + GTPBP7	This paper	N/A
HEK 293T Flp-In T-REx + GTPBP8	This paper	N/A
HEK 293T Flp-In T-REx + GTPBP10	This paper	N/A

HEK 293T Flp-In T-REx MRM1 KO	Kindly provided by Aaron D'Souza	N/A
HEK 293T Flp-In T-REx MRM2 KO	This paper	N/A
HEK 293T Flp-In T-REx MRM2 KO + empty vector	This paper	N/A
HEK 293T Flp-In T-REx MRM2 KO + MRM2 WT	This paper	N/A
HEK 293T Flp-In T-REx MRM2 KO + MRM2 K59A	This paper	N/A
HEK 293T Flp-In T-REx MRM2 KO + MRM2 D154A	This paper	N/A
HEK 293T Flp-In T-REx MRM2 KO + GTPBP5	This paper	N/A
HEK 293T Flp-In T-REx MRM2 KO + GTPBP7	This paper	N/A
HEK 293T Flp-In T-REx MRM2 KO + GTPBP8	This paper	N/A
HEK 293T Flp-In T-REx MRM2 KO + GTPBP10	This paper	N/A
HEK 293T Flp-In T-REx MRM3 KO	Kindly provided by Joanna Rorbach	N/A
D. melanogaster: GAL4 under act5C promoter y[1] w[*]; P{w[+mC]=Act5C-GAL4}25FO1/CyO, y[+]	BDSC	4414
D. melanogaster: GAL4 under da promoter w[*]; Kr[If-1]/CyO; P{w[+mW.hs]=GAL4-da.G32}UH1	BDSC	55850
D. melanogaster: GAL4 under Mef2 promoter y[1] w[*]; P{w[+mC]=GAL4-Mef2.R}3	BDSC	27390
D. melanogaster: GAL4 under nSyb promoter y[1] w[*]; P{w[+m*]=nSyb-GAL4.S}3	BDSC	51635
D. melanogaster: CG11447 RNAi w[1118]; P{GD7303}v16199	VDRC	v16199
D. melanogaster: lacZ RNAi w[1118]; P{GD936}v51446	VDRC	v51446
MRM2 Fw: ATCAGTTCACCTCCCCTCCT	Sigma-Aldrich	CKOZFND8984-1KT
MRM2 Rv: GAATCTGGTGCCTCTCGTTC	Sigma-Aldrich	CKOZFND8984-1KT
RNA adaptor 1: prArGrArUrCrGrGrArArGrArGrCrArCrArCrGrUrCddC	Sigma-Aldrich	N/A
RT primer: AGACGTGTGCTCTTCCG	Sigma-Aldrich	N/A
Adaptor 2: pAGATCGGAAGAGCGTCGTGTAG3ddC	Sigma-Aldrich	N/A
DmMRM2 Fw: TGGTCAAGGTGTGGGATAATG	Sigma-Aldrich	N/A
DmMRM2 Rv: CGTTGCTCCTTTAAAGTTCCTG	Sigma-Aldrich	N/A
αTub84B Fw: TGGGCCCGTCTGGACCACAA	Sigma-Aldrich	N/A
αTub84B Rv: TCGCCGTCACCGGAGTCCAT	Sigma-Aldrich	N/A
pWPXLD:IRES:PuroR	Kindly provided by Ester Perales-Clemente and Ricardo Marco-Lázaro	(Perales-Clemente *et al.*, 2008)
pWPXLD:IRES:PuroR MRM2 WT	This paper	N/A
pWPXLD:IRES:PuroR MRM2 K59A	This paper	N/A
pWPXLD:IRES:PuroR MRM2 D154A	This paper	N/A
pWPXLD:IRES:PuroR GTPBP5	This paper	N/A
pWPXLD:IRES:PuroR GTPBP7	This paper	N/A
pWPXLD:IRES:PuroR GTPBP8	This paper	N/A
pWPXLD:IRES:PuroR GTPBP10	This paper	N/A
psPAX2	Addgene	#12260
pMD2.G	Addgene	#12259

STAR aligner	(Dobin *et al.*, 2013)	https://github.com/alexdobin/STAR/releases
bam2ReadEnds.R	(Garcia-Campos *et al.*, 2019)	(García-Campos, 2019)
XCalibur 3.0.63	Thermo Scientific	N/A
Proteome Discoverer 1.4	Thermo Scientific	N/A
MaxQuant 1.5.8.3	Max Plank Institute of Biochemistry	https://www.maxquant.org/
Perseus	Max Plank Institute of Biochemistry	https://maxquant.net/perseus/
ImageJ	(Schindelin *et al.*, 2012)	https://imagej.nih.gov/ij/
FASTX-Toolkit	N/A	http://hannonlab.cshl.edu/fastx_toolkit/
bowtie, version 1	(Langmead *et al.*, 2009)	http://bowtie.cbcb.umd.edu/
RELION-3.1	(Zivanov *et al.*, 2018; Zivanov, Nakane and Scheres, 2020)	https://www3.mrc-lmb.cam.ac.uk/relion/
MotionCor2	(Zheng *et al.*, 2017)	http://msg.ucsf.edu/em/software/motioncor2.html
CTFFIND-4.1	(Rohou and Grigorieff, 2015)	http://grigoriefflab.janelia.org/ctf
cryoSPARC	(Punjani *et al.*, 2017)	https://cryosparc.com/
cryoEF	(Naydenova and Russo, 2017)	https://www.mrc-lmb.cam.ac.uk/crusso/cryoEF/
cryoDRGN	(Zhong *et al.*, 2021)	http://cb.csail.mit.edu/cb/cryodrgn/
Coot	(Emsley *et al.*, 2010)	https://www2.mrc-lmb.cam.ac.uk/personal/pemsley/coot/
PHENIX	(Adams *et al.*, 2010)	https://www.phenix-online.org/
MolProbity	(Chen *et al.*, 2010)	http://molprobity.biochem.duke.edu/
Chimera	(Pettersen *et al.*, 2004)	https://www.cgl.ucsf.edu/chimera/
ChimeraX	(Pettersen *et al.*, 2021)	https://www.cgl.ucsf.edu/chimerax/
OpenMS	(Röst *et al.*, 2016)	https://www.openms.de/
R	(R Core Team, 2014)	https://www.R-project.org/
NucleicAcidSearchEngine	(Wein *et al.*, 2020)	https://www.openms.de/comp/nase/
FeatureFinderID	(Weisser and Choudhary, 2017)	https://www.openms.de/
2200 TapeStation System	Agilent	N/A
Acclaim PepMap C18 reversed-phase column	Thermo Scientific	N/A
Acclaim PepMap C18 solid phase	Thermo Scientific	N/A
ÄKTAprime plus	GE Healthcare	N/A
Balch-type homogeniser	Isobiotec	N/A
Falcon 3EC Direct Electron Detector	FEI	N/A

Gradient Station	Biocomp	Part #153
HiSeq 4000 System	Illumina	N/A
Imager 680	Amersham	N/A
Incucyte ZOOM	Essen BioScience	N/A
NextSeq 500 System	Illumina	N/A
Nucleofector 2b Device	Lonza	AAB-1001
Optima MAX-XP Benchtop Ultracentrifuge	Beckman Coulter	N/A
Pellet pestles	Sigma-Aldrich	Z359971-1EA
ProXeon nanoLC1000 UPLC system	Thermo Scientific	N/A
Q Exactive HF mass spectrometer	Thermo Scientific	N/A
Q Exactive Plus electrospray mass spectrometer	Thermo Scientific	N/A
Quantifoil grid (Cu, 300 mesh, R2/2)	Agar Scientific	AGS173-2-100
QuantStudio 3 Real-Time PCR System	Applied Biosystems	A28567
Seahorse XF96 Extracellular Flux Analyzer	Agilent	N/A
Titan Krios	FEI	N/A
TLS-55 Swinging Bucket Rotor	Beckman Coulter	346936
Typhoon Biomolecular Imager	Amersham	N/A
Vitrobot Mark IV	FEI	N/A

Supplementary Table 3 | CryoEM map refinement and model validation statistics – consensus and intersubunit interface focused classification with signal subtraction.

	Consensus	Intersubunit interface (FCwSS)				
		1	2	3	4	5
**Data processing**						
Number of particles	1,191,870	119,341	85,728	748,427	224,933	13,441
Symmetry imposed	C_1	C_1	C_1	C_1	C_1	C_1
Map resolution (Å)	2.58	3.08	3.15	2.89	2.89	4.19
FSC threshold	0.143	0.143	0.143	0.143	0.143	0.143
Minimum (Å)	2.27	2.72	2.84	2.50	2.53	3.30
Median (Å)	2.94	3.85	4.05	3.37	3.32	5.64
Maximum (Å)	11.13	17.52	15.63	12.96	12.62	23.21
Map sharpening *B* factor (Å^2)		-83.0568	-86.8049	-93.5455	-76.9201	-85.3014
**Refinement**						
Initial model used		5OOL			5OOL	
Model resolution range (Å)		7.2 – 2.8			7.2 – 2.8	
Model composition						
Nonhydrogen atoms		155,332			157,957	
Protein residues		7,113			7,020	
RNA bases		1,196			1,317	
B factors (mean, Å^2)						
Protein residues		39			15	
RNA residues		73			19	
Ligands		31			11	
R.m.s. deviations						
Bond lengths (Å)		0.005			0.002	
Bond angles (°)		0.498			0.548	
Model validation						
MolProbity score (percentile, 0-99 Å)		1.56 (94)			1.68 (90)	
Clashscore (all atoms)		6.01			6.24	
Poor rotamers (%)		0.73			0.34	
Ramachandran plot						
Favoured (%)		96.51			95.17	
Allowed (%)		3.49			4.77	
Disallowed (%)		0.00			0.06	
CaBLAM outliers (%)		2.0			2.5	
**Model vs. Map validation**						
CC_{mask}		0.60			0.65	
CC_{box}		0.65			0.65	
CC_{volume}		0.62			0.67	
FSC model-map		3.5			3.1	
FSC threshold		0.5			0.5	
**RNA**						
Correct sugar puckers (%)		98.58			99.32	
Good backbone conformations (%)		73.83			76.31	
**Data deposition**						
EMDB		EMD-13965	EMD-13962	EMD-13963	EMD-13967	EMD-13966
PDB		7QH6			7QH7	

Supplementary Table 4 | CryoEM map refinement – anti-association module and central protuberance focused classification with signal subtraction.

	Anti-association module (FCwSS)		Central protuberance (FCwSS)		
	1	2	1	2	3
**Data processing**					
Number of particles	194,162	997,693	1,012,600	91,905	87,365
Symmetry imposed	C_1	C_1	C_1	C_1	C_1
Map resolution (Å)	3.03	2.77	2.77	3.15	3.15
FSC threshold	0.143	0.143	0.143	0.143	0.143
Minimum (Å)	2.73	2.42	2.43	2.80	2.83
Median (Å)	3.64	3.16	3.16	3.90	3.99
Maximum (Å)	15.92	11.77	11.53	16.80	16.90
