# Supplementary material for: A late-stage assembly checkpoint of the human mitochondrial ribosome large subunit
Source: Nat Commun. 2022 Feb 17;13:929. doi: 10.1038/s41467-022-28503-5 (PMC8854578; doi:10.1038/s41467-022-28503-5)
Supplement: Supplementary file 3 — Description of Additional Supplementary Files [file 41467_2022_28503_MOESM3_ESM.pdf]

Description of Additional Supplementary Files

File name: Supplementary Data 1

Description: Code for calculating RT-stop scores and cleavage protection scores.

File name: Supplementary Data 2

Description: Quantitative density gradient analysis by mass spectrometry (qDGMS) of mitochondrial lysates from parental and MRM2 knock-out cells.

File name: Supplementary Movie 1

Description: Inspection of structural heterogeneity in mtLSU particles in the absence of MRM2. UMAP visualisation of the latent space is presented on the right, with the on-data cluster centres represented as diamonds. The latent space exploration path though cluster centres (in no particular order) can be followed by a red dot, which corresponds to the volume (latent space representation) shown on the left. All volumes are presented with the same isosurface threshold.
