# Supplementary material for: A late-stage assembly checkpoint of the human mitochondrial ribosome large subunit
Source: Nat Commun. 2022 Feb 17;13:929. doi: 10.1038/s41467-022-28503-5 (PMC8854578; doi:10.1038/s41467-022-28503-5)
Supplement: Supplementary file 7 — Reporting Summary [file 41467_2022_28503_MOESM7_ESM.pdf]

nature portfolio

Corresponding author(s): Michal Minczuk

Last updated by author(s): Aug 2, 2021

Reporting Summary

Nature Portfolio wishes to improve the reproducibility of the work that we publish. This form provides structure for consistency and transparency in reporting. For further information on Nature Portfolio policies, see our Editorial Policies and the Editorial Policy Checklist.

Statistics

For all statistical analyses, confirm that the following items are present in the figure legend, table legend, main text, or Methods section.

n/a	Confirmed	
☐	☒	The exact sample size (*n*) for each experimental group/condition, given as a discrete number and unit of measurement
☐	☒	A statement on whether measurements were taken from distinct samples or whether the same sample was measured repeatedly
☐	☒	The statistical test(s) used AND whether they are one- or two-sided<br>*Only common tests should be described solely by name; describe more complex techniques in the Methods section.*
☒	☐	A description of all covariates tested
☒	☐	A description of any assumptions or corrections, such as tests of normality and adjustment for multiple comparisons
☐	☒	A full description of the statistical parameters including central tendency (e.g. means) or other basic estimates (e.g. regression coefficient) AND variation (e.g. standard deviation) or associated estimates of uncertainty (e.g. confidence intervals)
☐	☒	For null hypothesis testing, the test statistic (e.g. *F*, *t*, *r*) with confidence intervals, effect sizes, degrees of freedom and *P* value noted<br>*Give P values as exact values whenever suitable.*
☒	☐	For Bayesian analysis, information on the choice of priors and Markov chain Monte Carlo settings
☒	☐	For hierarchical and complex designs, identification of the appropriate level for tests and full reporting of outcomes
☒	☐	Estimates of effect sizes (e.g. Cohen's *d*, Pearson's *r*), indicating how they were calculated

Our web collection on statistics for biologists contains articles on many of the points above.

Software and code

Policy information about availability of computer code

Data collection	Thermo Fisher EPU software v2.7
Data analysis	STAR aligner (Dobin et al., 2013)<br>https://github.com/alexdobin/STAR/releases<br>bam2ReadEnds.R (Garcia-Campos et al., 2019)<br>(García-Campos, 2019)<br>XCalibur 3.0.63 Thermo Scientific N/A<br>Proteome Discoverer 1.4 Thermo Scientific N/A<br>MaxQuant 1.5.8.3 Max Plank Institute of Biochemistry https://www.maxquant.org/<br>Perseus Max Plank Institute of Biochemistry https://maxquant.net/perseus/<br>ImageJ (Schindelin et al., 2012)<br>https://imagej.nih.gov/ij/<br>FASTX-Toolkit N/A http://hannonlab.cshl.edu/fastx_toolkit/<br>bowtie, version 1 (Langmead et al., 2009)<br>http://bowtie.cbcb.umd.edu/<br>RELION-3.1 (Zivanov et al., 2018; Zivanov, Nakane and Scheres, 2020)<br>https://www3.mrc-lmb.cam.ac.uk/relion/<br>MotionCor2 (Zheng et al., 2017)<br>http://msg.ucsf.edu/em/software/motioncor2.html<br>CTFFIND-4.1 (Rohou and Grigorieff, 2015)<br>http://grigoriefflab.janelia.org/ctf<br>cryoSPARC (Punjani et al., 2017)<br>https://cryosparc.com/

cryoEF (Naydenova and Russo, 2017)
https://www.mrc-lmb.cam.ac.uk/crusso/cryoEF/
cryoDRGN (Zhong et al., 2021)
http://cb.csail.mit.edu/cb/cryodrgn/
Coot (Emsley et al., 2010)
https://www2.mrc-lmb.cam.ac.uk/personal/pemsley/coot/
PHENIX (Adams et al., 2010)
https://www.phenix-online.org/
MolProbity (Chen et al., 2010)
http://molprobity.biochem.duke.edu/
Chimera (Pettersen et al., 2004)
https://www.cgl.ucsf.edu/chimera/
ChimeraX (Pettersen et al., 2021)
https://www.cgl.ucsf.edu/chimerax/
OpenMS (Röst et al., 2016)
https://www.openms.de/
R (R Core Team, 2014)
https://www.R-project.org/
NucleicAcidSearchEngine (Wein et al., 2020)
https://www.openms.de/comp/nase/
FeatureFinderID (Weisser and Choudhary, 2017)
https://www.openms.de/
Incucyte ZOOM software
Other scripts used in this study are provided in full with the manuscript.

For manuscripts utilizing custom algorithms or software that are central to the research but not yet described in published literature, software must be made available to editors and reviewers. We strongly encourage code deposition in a community repository (e.g. GitHub). See the Nature Portfolio guidelines for submitting code & software for further information.

Data

Policy information about availability of data

All manuscripts must include a data availability statement. This statement should provide the following information, where applicable:

- Accession codes, unique identifiers, or web links for publicly available datasets
- A description of any restrictions on data availability
- For clinical datasets or third party data, please ensure that the statement adheres to our policy

Sequencing data generated in this study have been deposited in the GEO and ArrayExpress databases under accession codes GSE179085 and E-MTAB-11292, respectively. CryoEM maps generated in this study have been deposited in the EMDB database under accession codes EMD-13965, EMD-13962, EMD-13963, EMD-13967 and EMD-13966. Atomic models generated in this study have been deposited in the PDB database under accession codes 7QH6 and 7QH7. The source data underlying all figures and supplementary figures are provided as a Source Data file. Any additional information required to reanalyse the data reported in this paper is available from the lead contact upon request. Publicly available published datasets used in this study are listed in Supplementary Table 2.

Field-specific reporting

Please select the one below that is the best fit for your research. If you are not sure, read the appropriate sections before making your selection.

☒ Life sciences ☐ Behavioural & social sciences ☐ Ecological, evolutionary & environmental sciences

For a reference copy of the document with all sections, see nature.com/documents/nr-reporting-summary-flat.pdf

Life sciences study design

All studies must disclose on these points even when the disclosure is negative.

Sample size	Sample size was chosen to achieve a n at least of 3 and whenever experimentally possible and reasonable, more replicates were performed, as mentioned throughout the manuscript, and described in the legend of the appropriate figures. This is based in our previous experience while performing the methodologies described in this study.
Data exclusions	No data was excluded from analysis.
Replication	Experiments were replicated as mentioned throughout the manuscript, and described in the legend of the appropriate figures.
Randomization	The methodologies applied in this study do not require sample randomization.
Blinding	The methodologies applied in this study do not require a blind study.

Reporting for specific materials, systems and methods

We require information from authors about some types of materials, experimental systems and methods used in many studies. Here, indicate whether each material, system or method listed is relevant to your study. If you are not sure if a list item applies to your research, read the appropriate section before selecting a response.

Materials & experimental systems

n/a	Involved in the study
☐	☒ Antibodies
☐	☒ Eukaryotic cell lines
☒	☐ Palaeontology and archaeology
☐	☒ Animals and other organisms
☒	☐ Human research participants
☒	☐ Clinical data
☒	☐ Dual use research of concern

Methods

n/a	Involved in the study
☒	☐ ChIP-seq
☒	☐ Flow cytometry
☒	☐ MRI-based neuroimaging

Antibodies

Antibodies used	Mouse monoclonal anti-β-actin Sigma-Aldrich A2228 1:50000<br>Rabbit polyclonal anti-GTPBP5 Atlas Antibodies HPA047379 1:1000<br>Rabbit polyclonal anti-GTPBP7 Atlas Antibodies HPA037827 1:1000<br>Rabbit polyclonal anti-GTPBP8 Atlas Antibodies HPA034831 1:1000<br>Rabbit polyclonal anti-GTPBP10 Atlas Antibodies HPA021076 1:1000<br>Rabbit polyclonal anti-MRM1 Atlas Antibodies HPA021598 1:1000<br>Mouse monoclonal anti-MRM2 MyBioSource MBS120390 1:100<br>Rabbit polyclonal anti-MRM3 Atlas Antibodies HPA022534 1:1000<br>Mouse monoclonal anti-MT-CO1 Abcam ab14705 1:1000<br>Mouse monoclonal anti-MT-CO2 Abcam ab110258 1:1000<br>Rabbit polyclonal anti-MTRES1 Atlas Antibodies HPA049535 1:1000<br>Mouse monoclonal anti-SDHB Abcam ab14714 1:1000<br>Mouse monoclonal anti-TOM22 Abcam ab10436 1:4000<br>Rabbit polyclonal anti-uL3m Proteintech 16584-1-AP 1:1000<br>Rabbit polyclonal anti-uS17m Proteintech 18881-1-AP 1:1000<br>Mouse monoclonal anti-Vinculin Sigma-Aldrich V4505 1:10000<br>Rabbit polyclonal anti-COX4 Kindly provided by Edward Owusu-Ansah (Murari et al., 2020) 1:1000<br>Rabbit polyclonal anti-mt:ND1 Kindly provided by Edward Owusu-Ansah (Murari et al., 2020) 1:1000<br>Mouse monoclonal anti-PDHA1 Abcam ab110334 1:1000<br>Rabbit polyclonal anti-SDHA Kindly provided by Edward Owusu-Ansah (Murari et al., 2020) 1:1000<br>Rabbit polyclonal anti-UQCR-C2 Kindly provided by Edward Owusu-Ansah (Murari et al., 2020) 1:1000<br>Goat anti-mouse IgG (H+L), HRP Conjugate Promega W4021 1:3000<br>Goat anti-rabbit IgG (H+L), HRP Conjugate Promega W4011 1:2000
Validation	anti-MRM1, anti-MRM2 and anti-MRM3 were validated using knock-out cell lines generated in our laboratory. Other antibodies were validated by the supplier (please refer to the manufacturer's notes on their website).

Eukaryotic cell lines

Policy information about cell lines

Cell line source(s)	HEK Flp-In T-REx 293 Thermo Fisher Scientific
Authentication	Cell lines were authenticated by the supplier (please refer to the supplier's website).
Mycoplasma contamination	Cell medium was tested for mycoplasma contamination. All tests were negative.
Commonly misidentified lines<br>(See ICLAC register)	No commonly misidentified cell lines were used in this study.

Animals and other organisms

Policy information about studies involving animals; ARRIVE guidelines recommended for reporting animal research

Laboratory animals	Drosophila melanogaster. Age and gender of studied animals is described where appropriate throughout the main text.
Wild animals	The study did not involve wild animals.
Field-collected samples	The study did not involve samples collected from the field.

Ethics oversight | No ethical approval was needed.

Note that full information on the approval of the study protocol must also be provided in the manuscript.
