# Supplementary figures and images for: A late-stage assembly checkpoint of the human mitochondrial ribosome large subunit
Source: Nat Commun. 2022 Feb 17;13:929. doi: 10.1038/s41467-022-28503-5 (PMC8854578; doi:10.1038/s41467-022-28503-5)

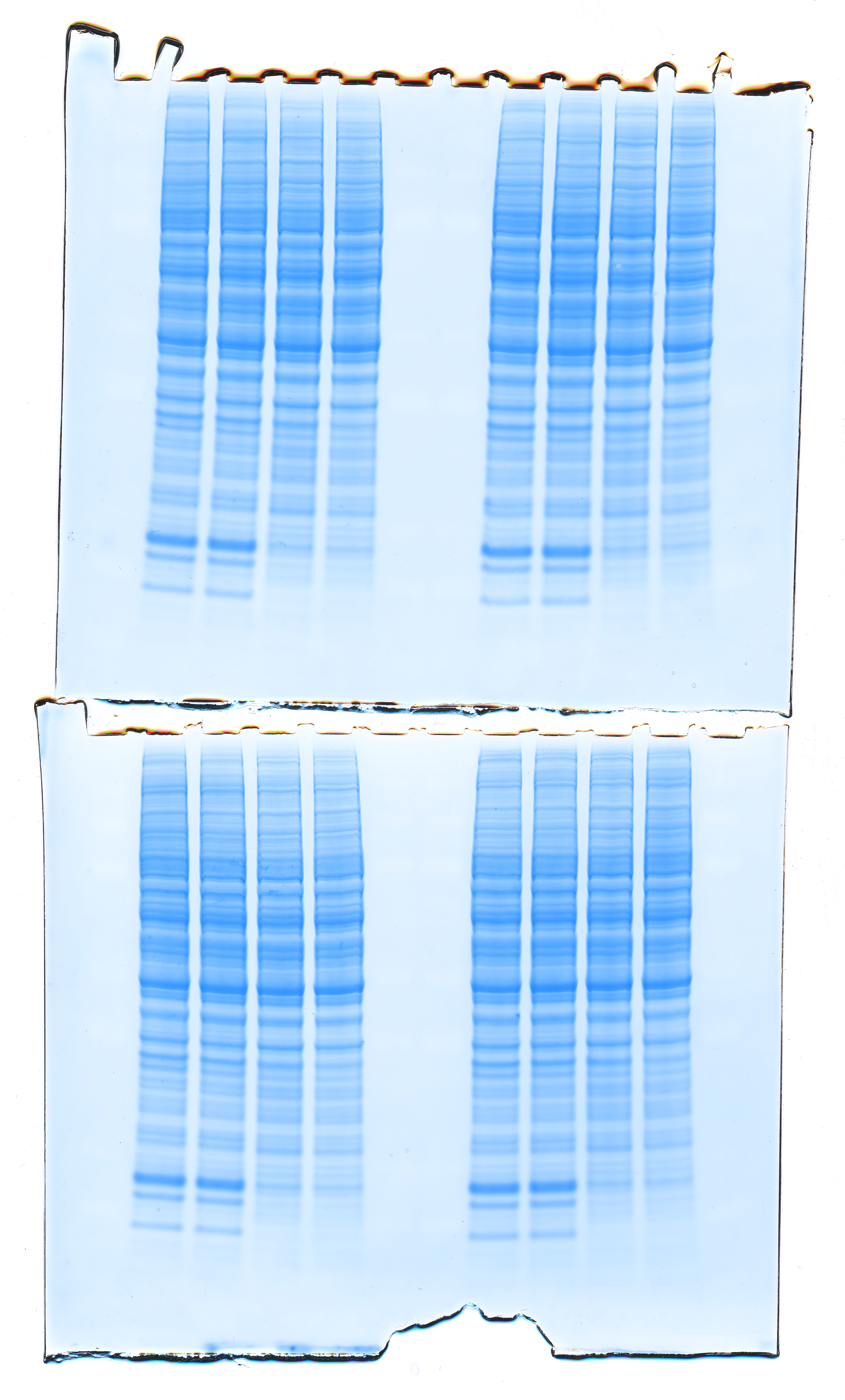

Supplement: Supplementary file 8 — Source Data [file 41467_2022_28503_MOESM8_ESM.zip › FigS1_CBB.tif]

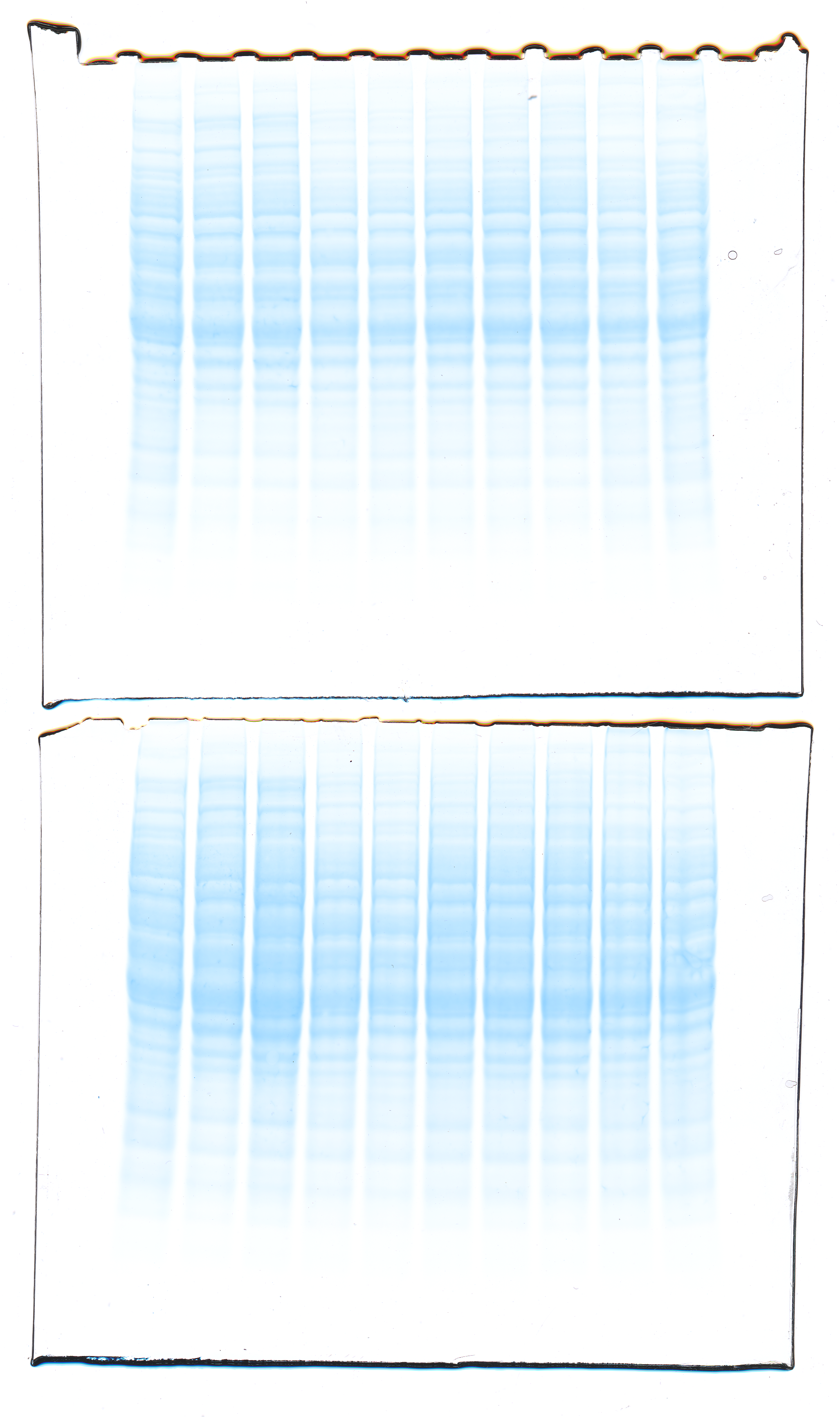

Supplement: Supplementary file 8 — Source Data [file 41467_2022_28503_MOESM8_ESM.zip › Fig6c_CBB.tif]

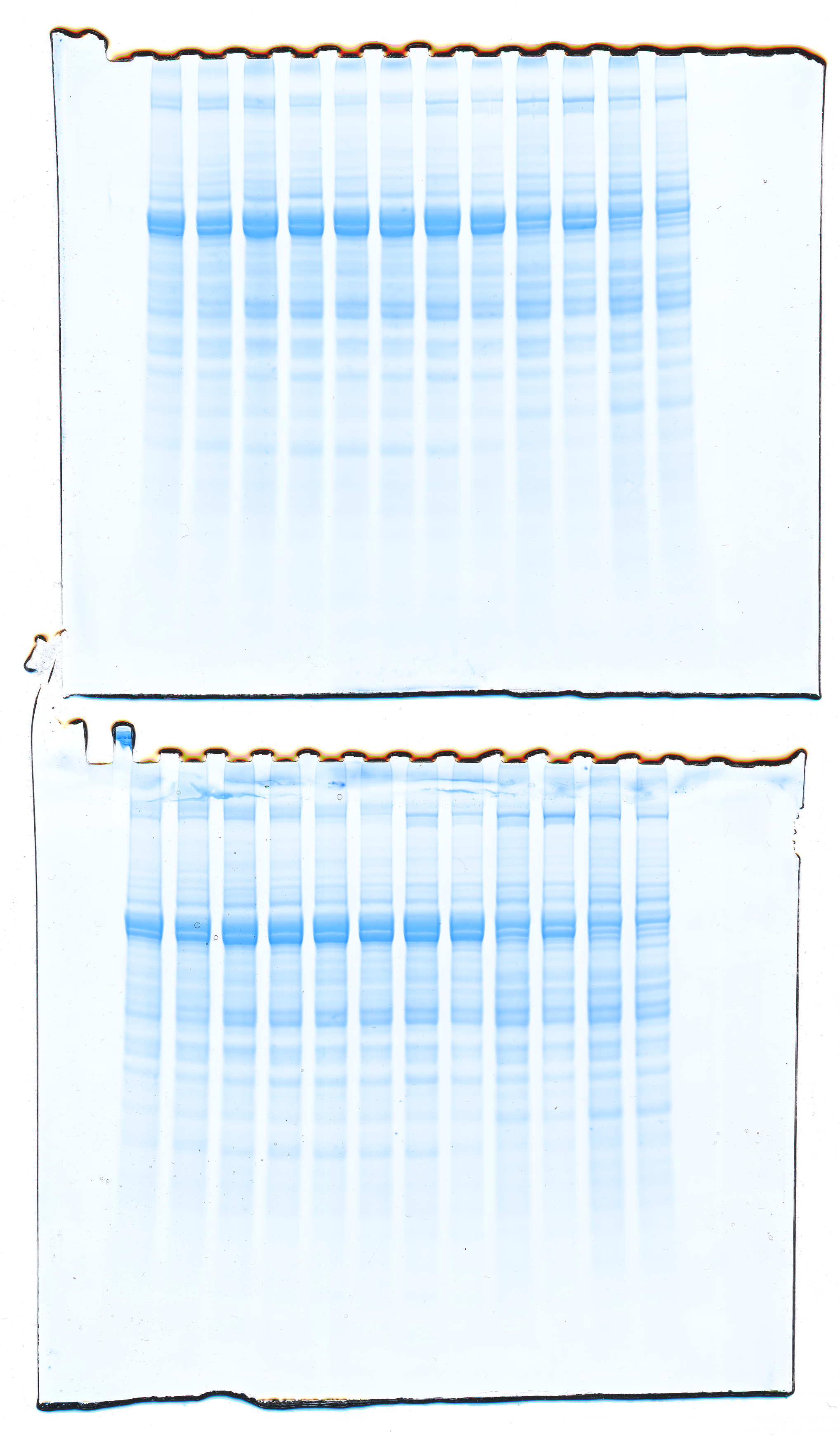

Supplement: Supplementary file 8 — Source Data [file 41467_2022_28503_MOESM8_ESM.zip › Fig7c_CBB.tif]

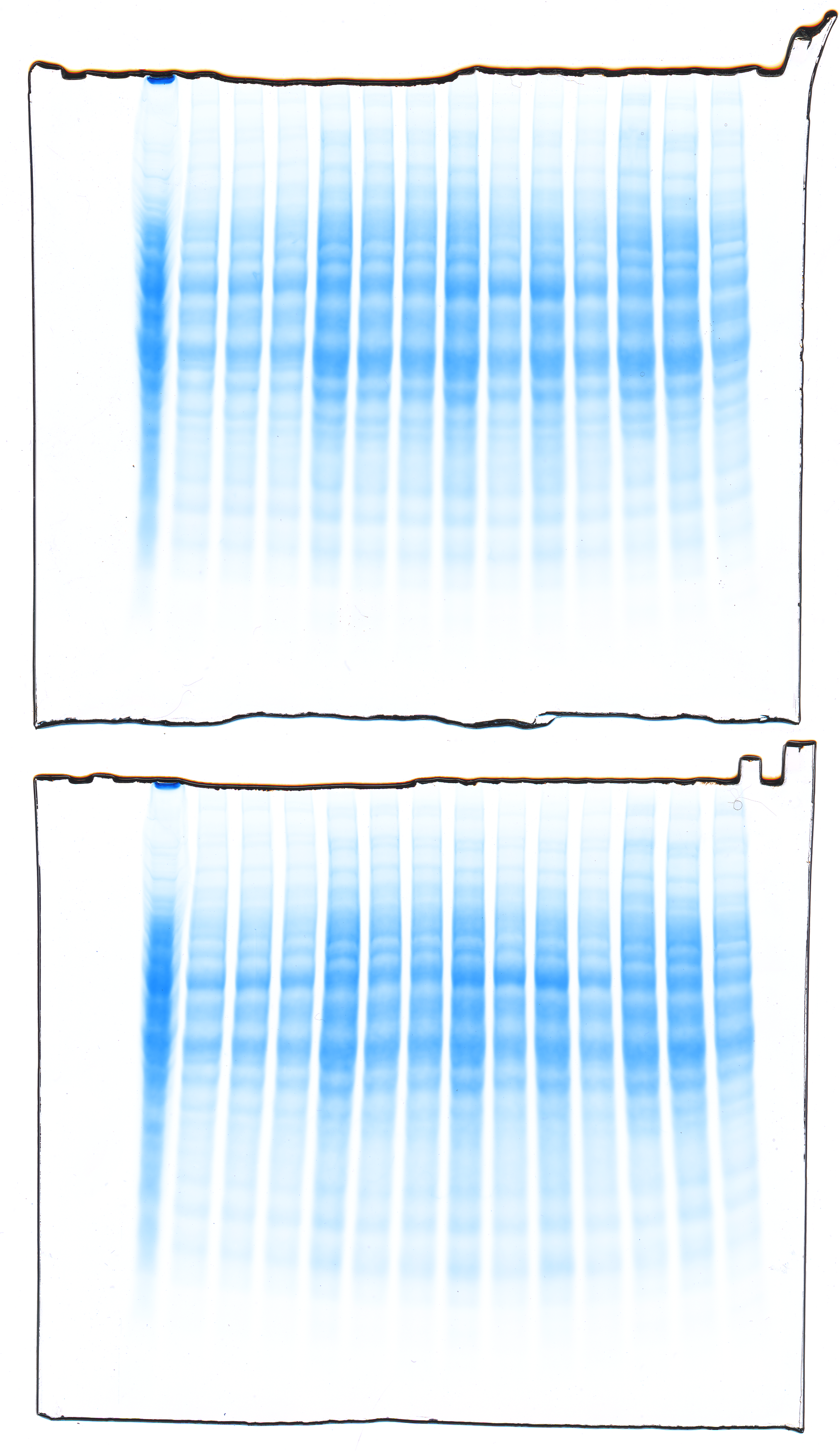

Supplement: Supplementary file 8 — Source Data [file 41467_2022_28503_MOESM8_ESM.zip › FigS11_CBBmirror.tif]

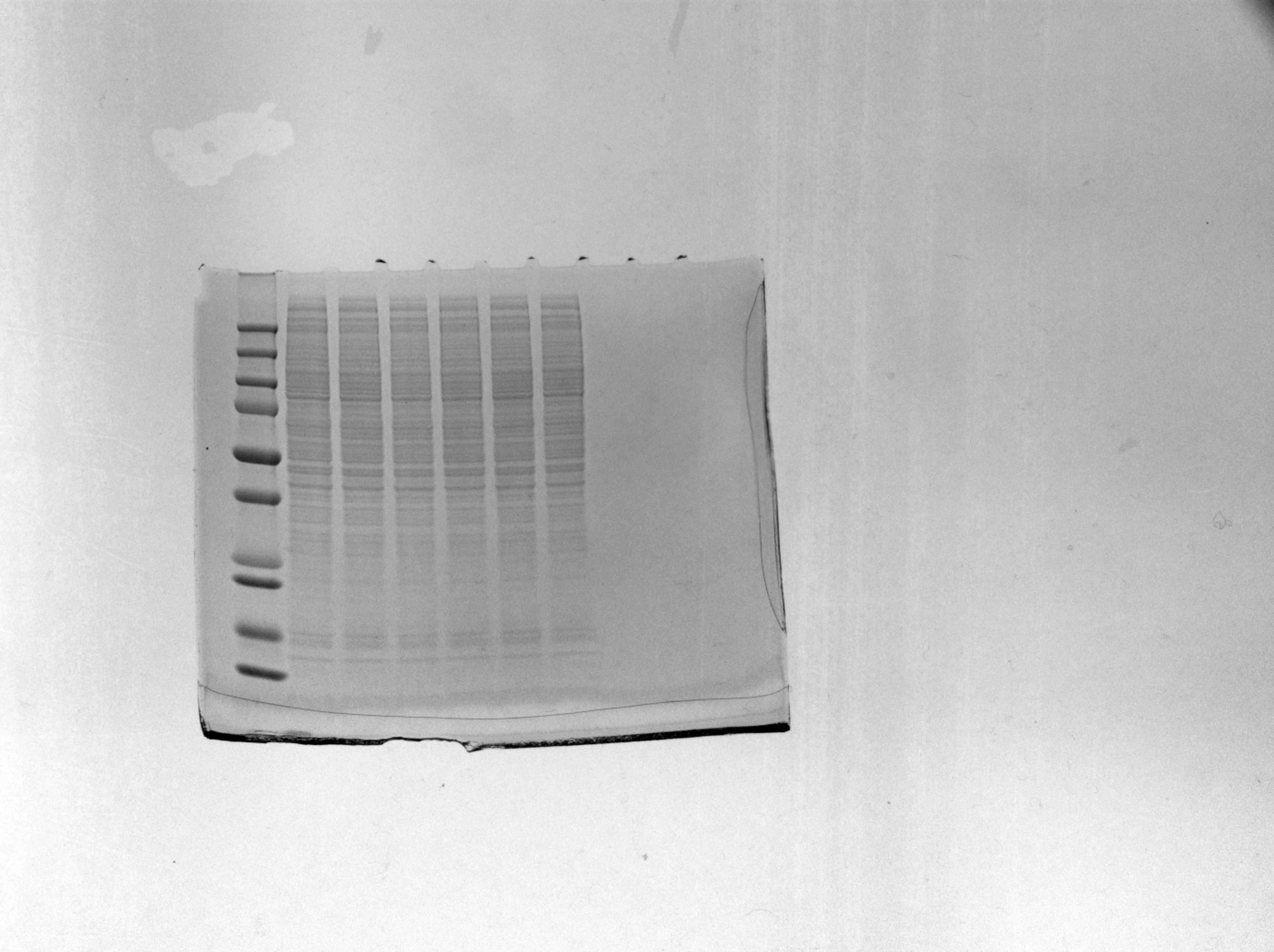

Supplement: Supplementary file 8 — Source Data [file 41467_2022_28503_MOESM8_ESM.zip › Fig3b_CBB.tif]

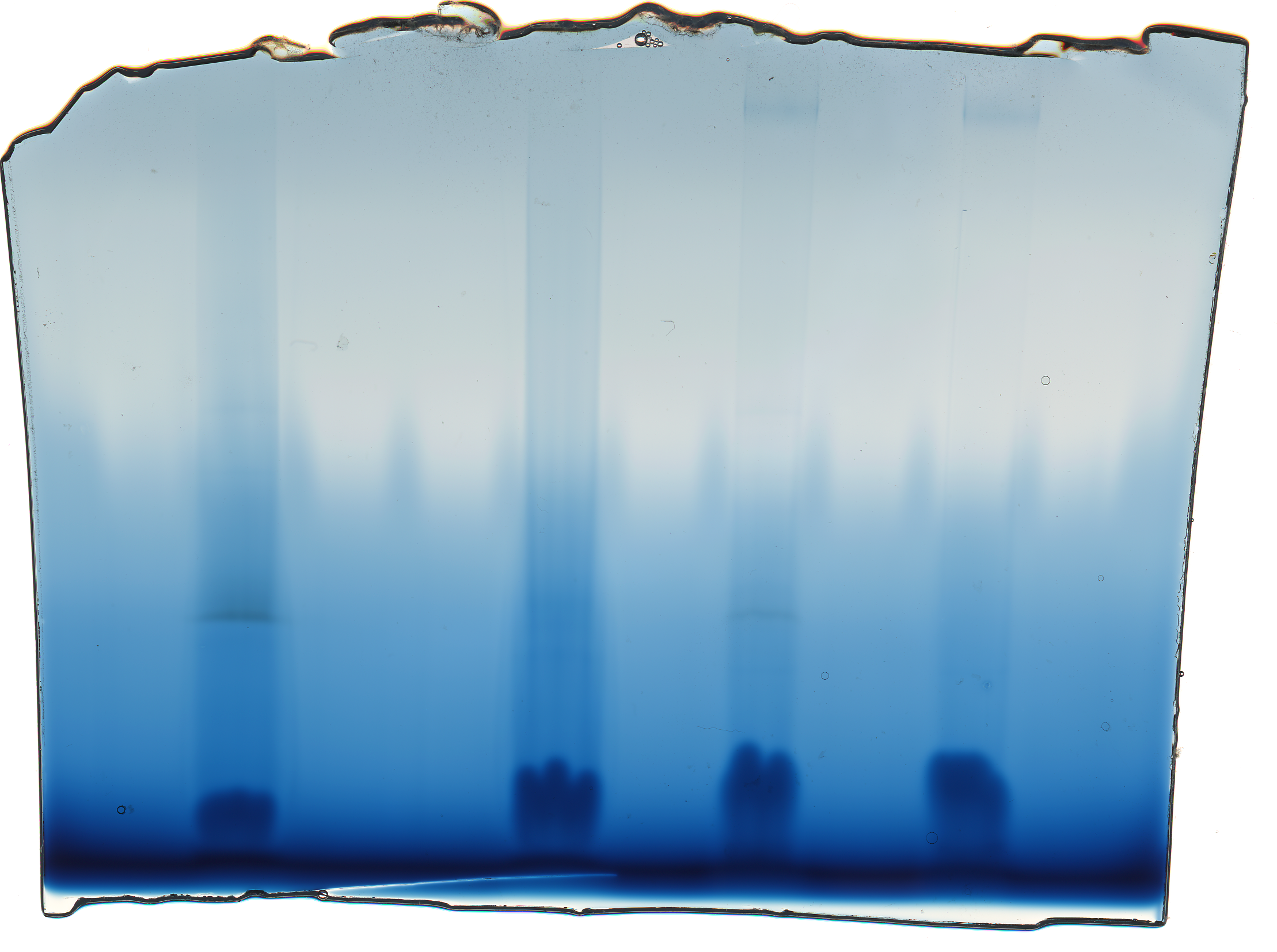

Supplement: Supplementary file 8 — Source Data [file 41467_2022_28503_MOESM8_ESM.zip › FigS2c_CIV.tif]

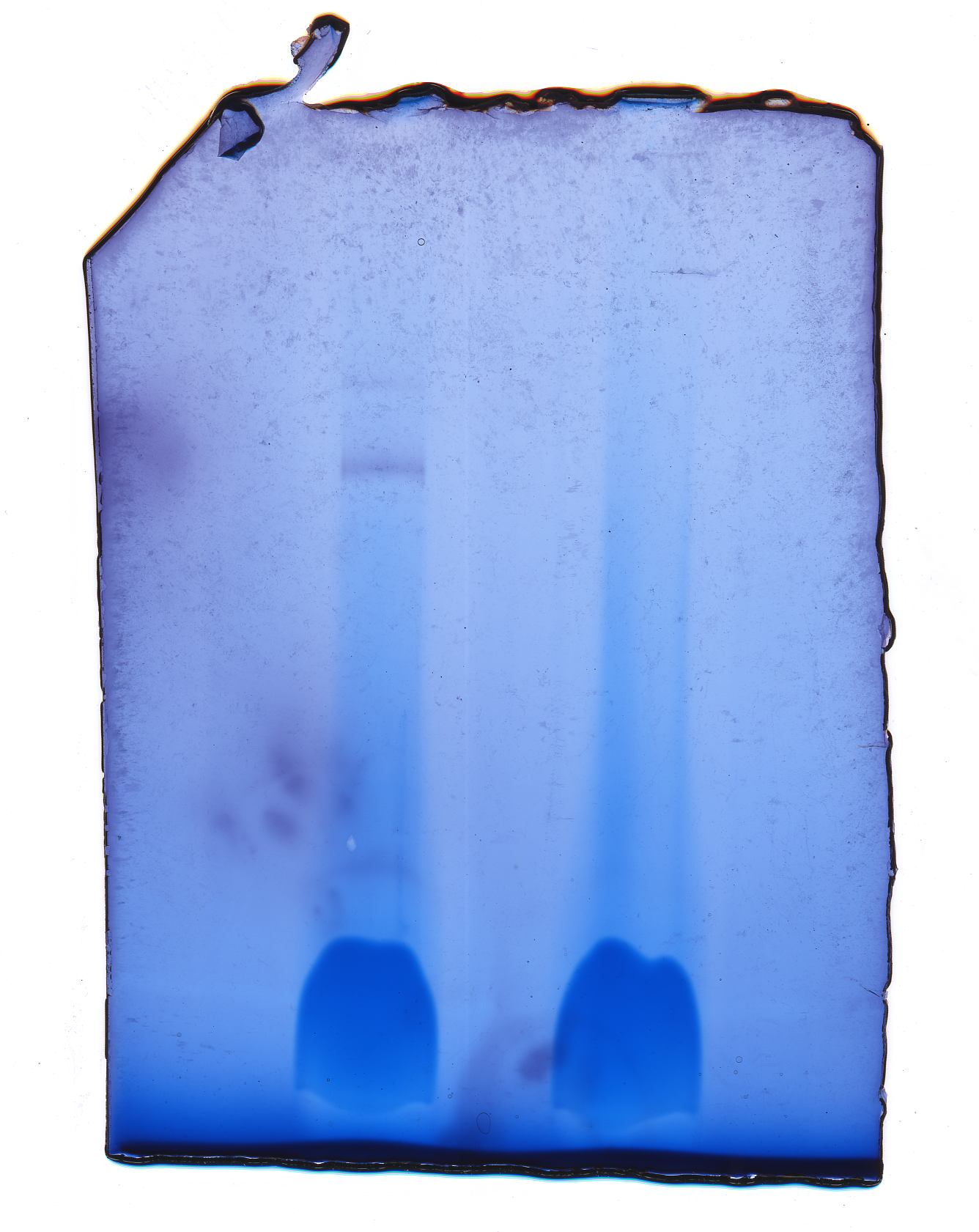

Supplement: Supplementary file 8 — Source Data [file 41467_2022_28503_MOESM8_ESM.zip › FigS2c_CI.tif]

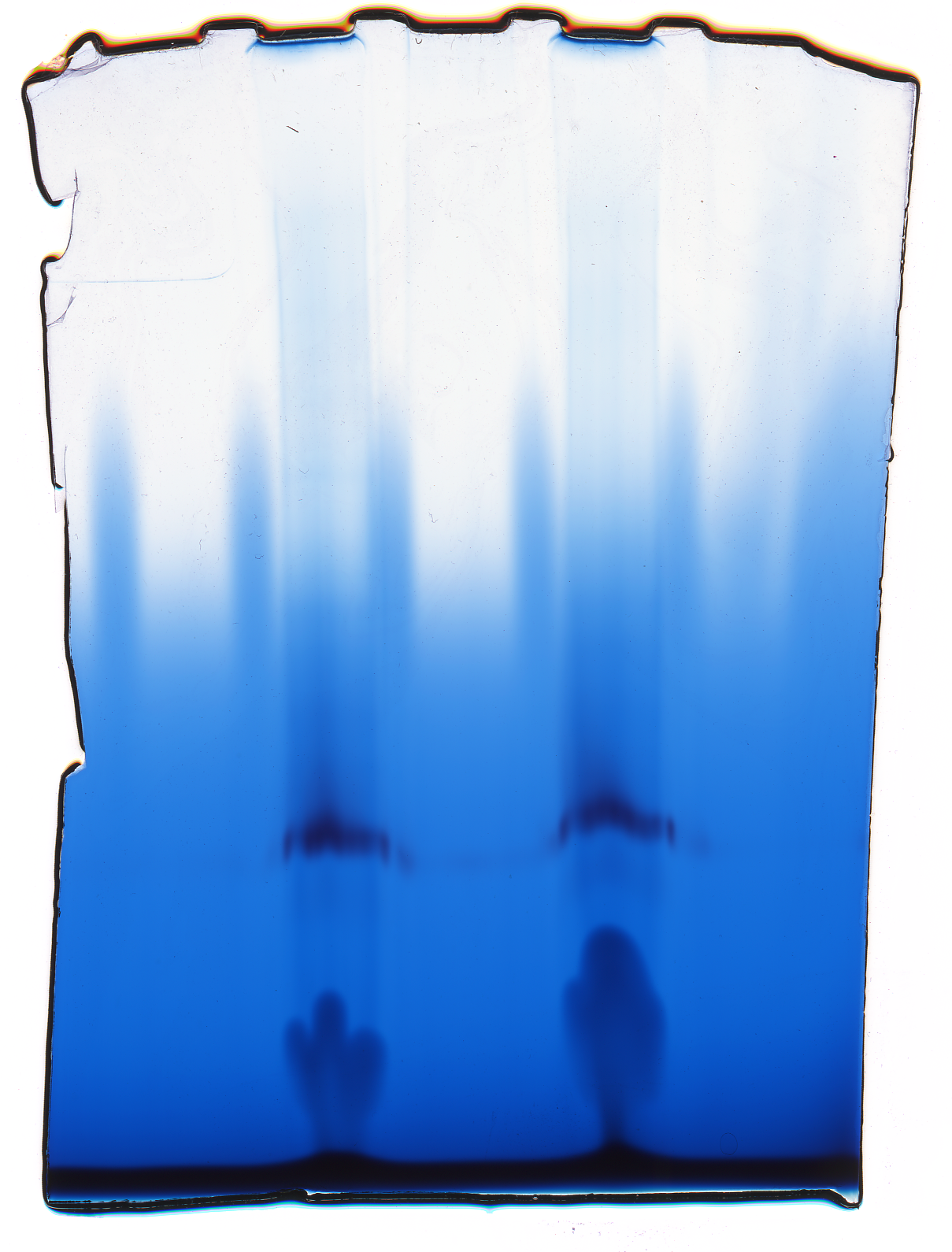

Supplement: Supplementary file 8 — Source Data [file 41467_2022_28503_MOESM8_ESM.zip › FigS2c_CII.tif]

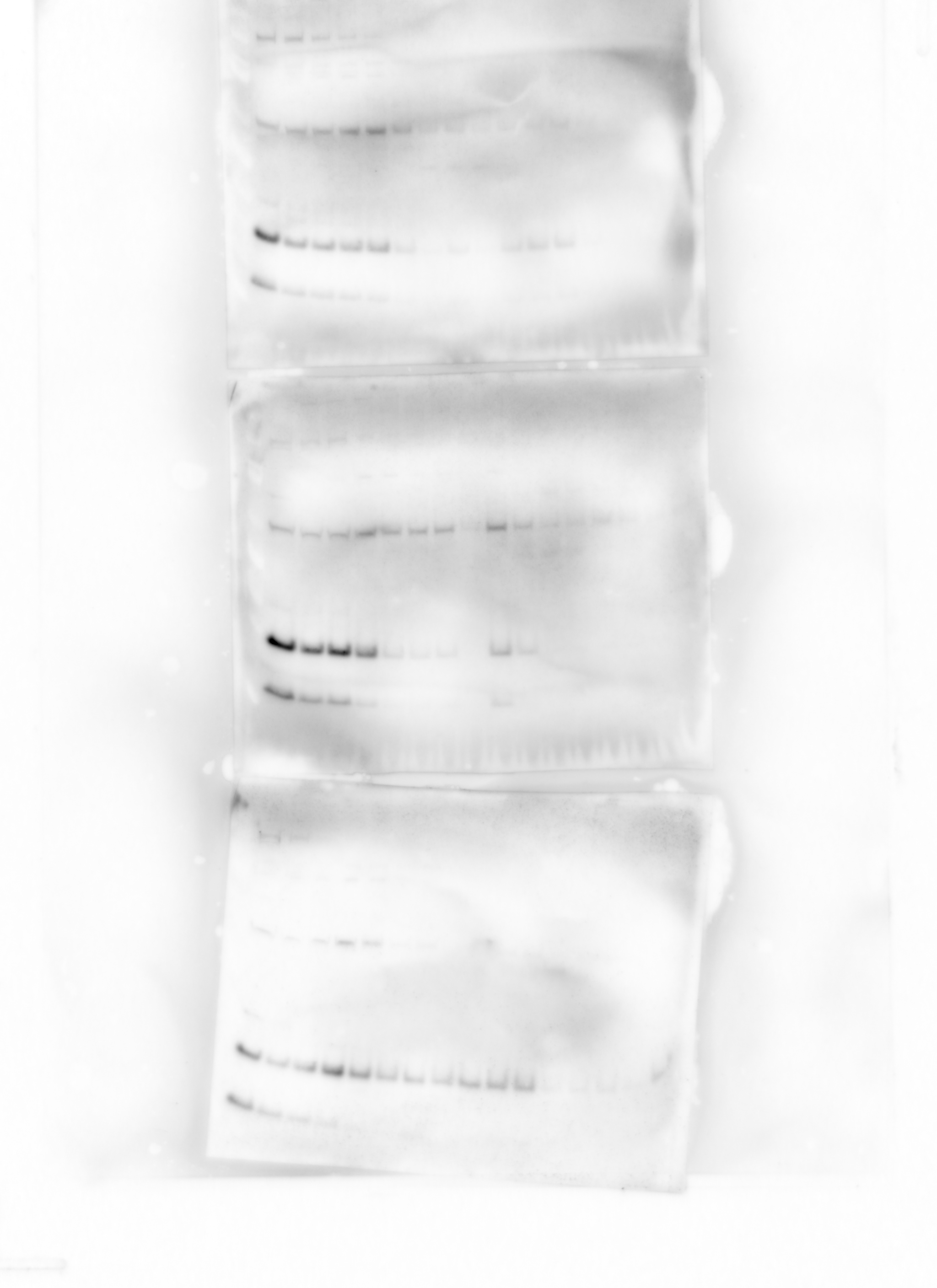

Supplement: Supplementary file 8 — Source Data [file 41467_2022_28503_MOESM8_ESM.zip › FigS6_MTRES1.tif]

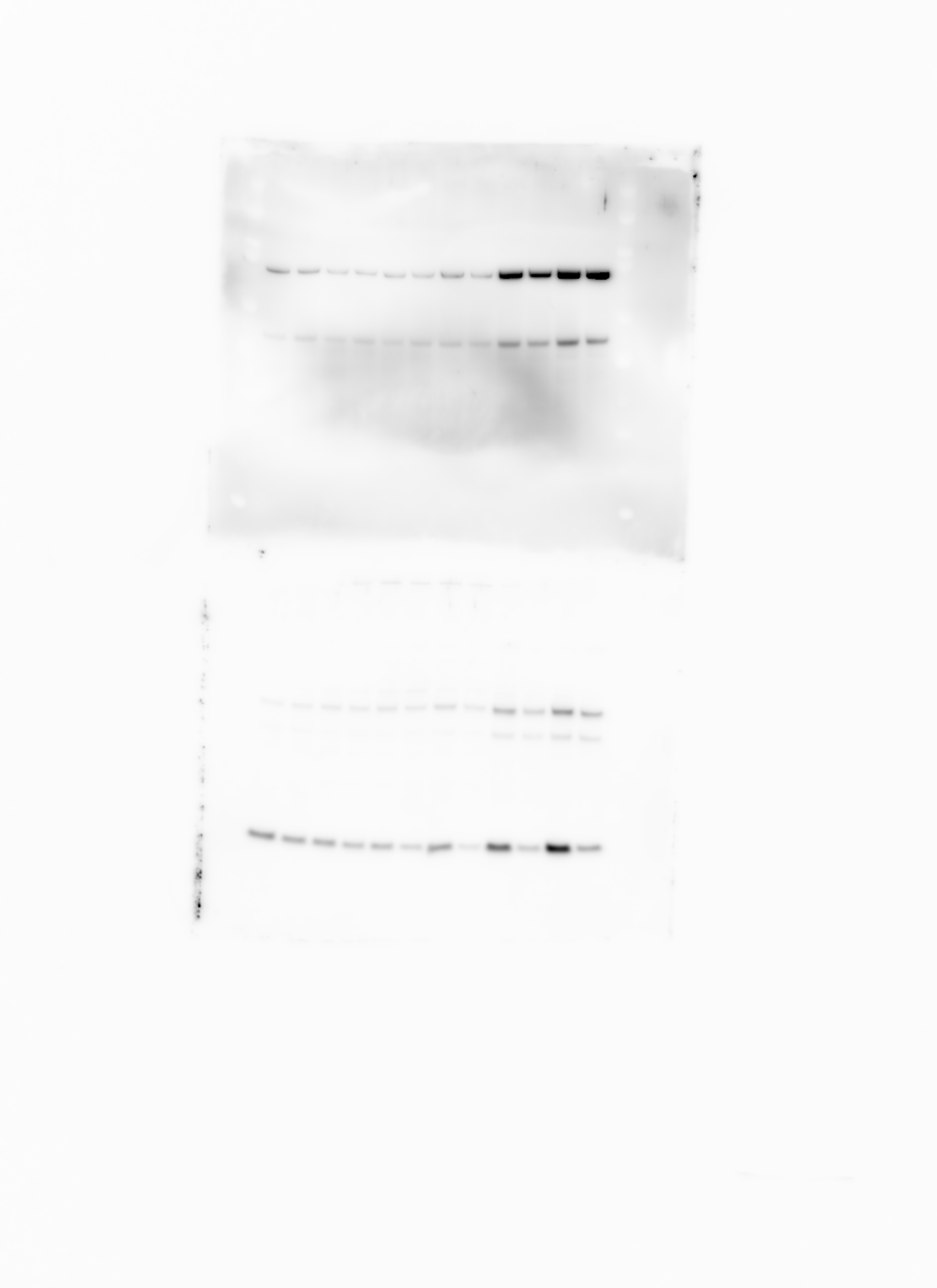

Supplement: Supplementary file 8 — Source Data [file 41467_2022_28503_MOESM8_ESM.zip › Fig7c_SDHA_PDHA.tif]

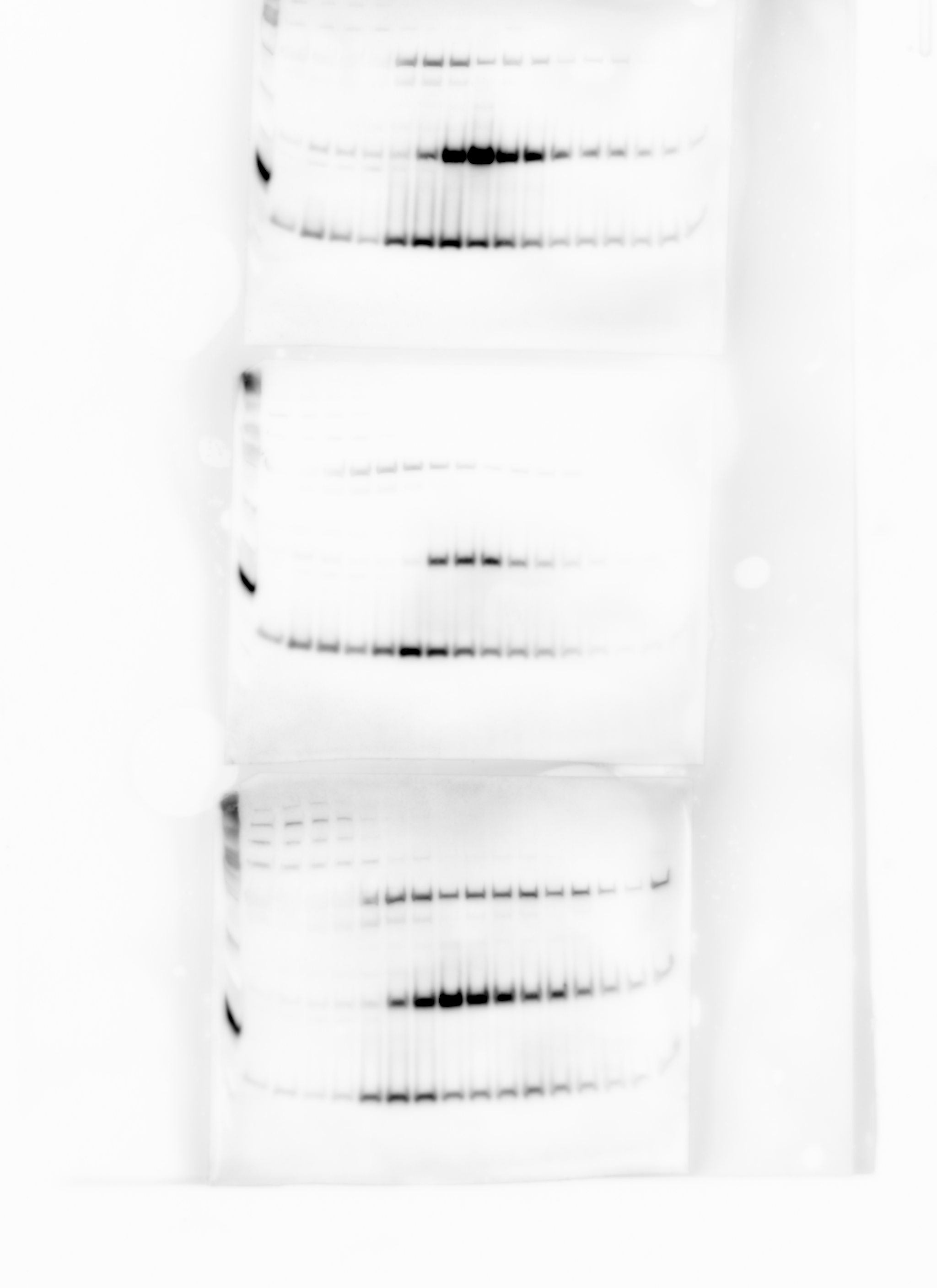

Supplement: Supplementary file 8 — Source Data [file 41467_2022_28503_MOESM8_ESM.zip › FigS6_uL3m_uS17m.tif]

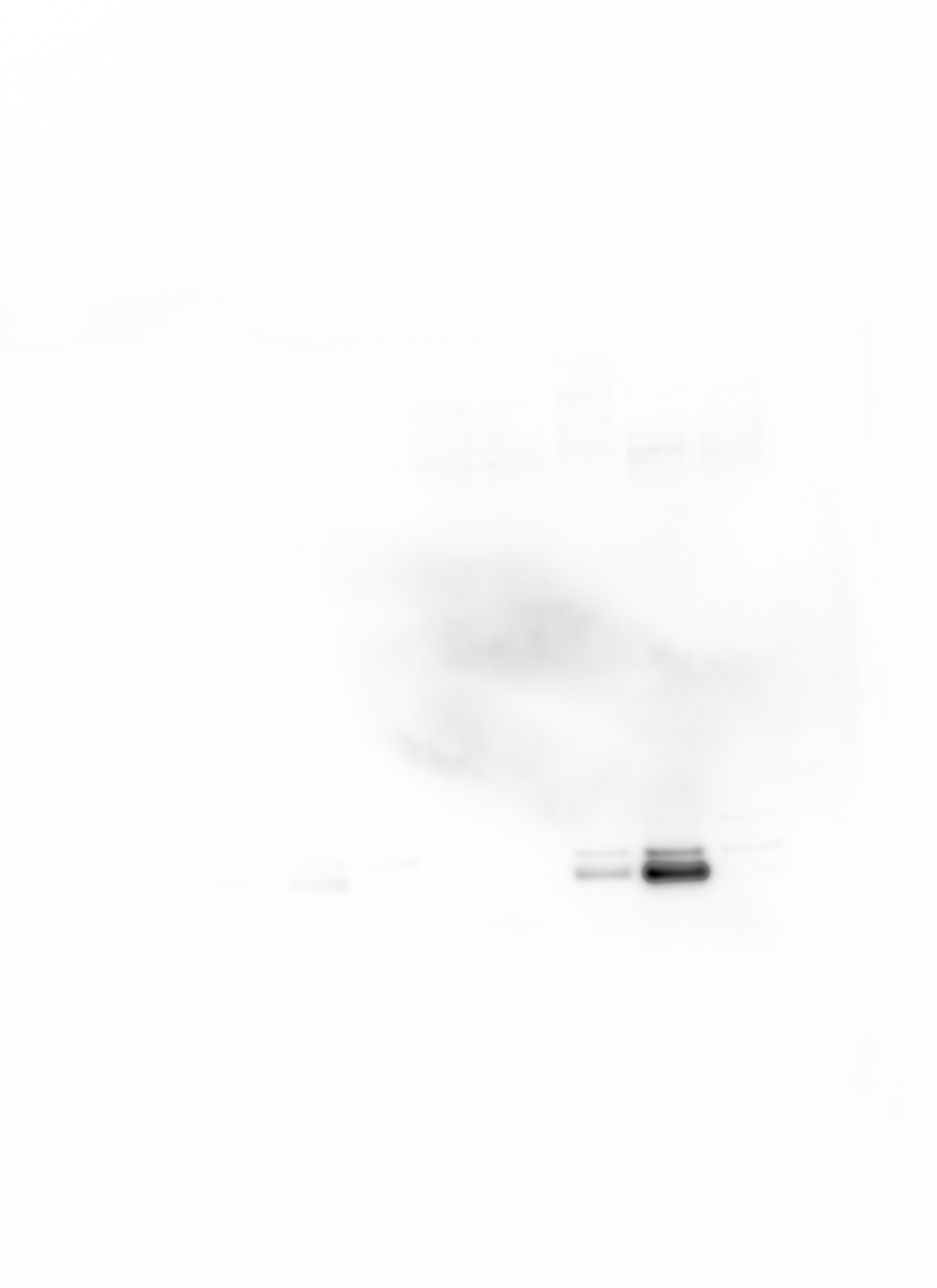

Supplement: Supplementary file 8 — Source Data [file 41467_2022_28503_MOESM8_ESM.zip › Fig6c_MRM2.tif]

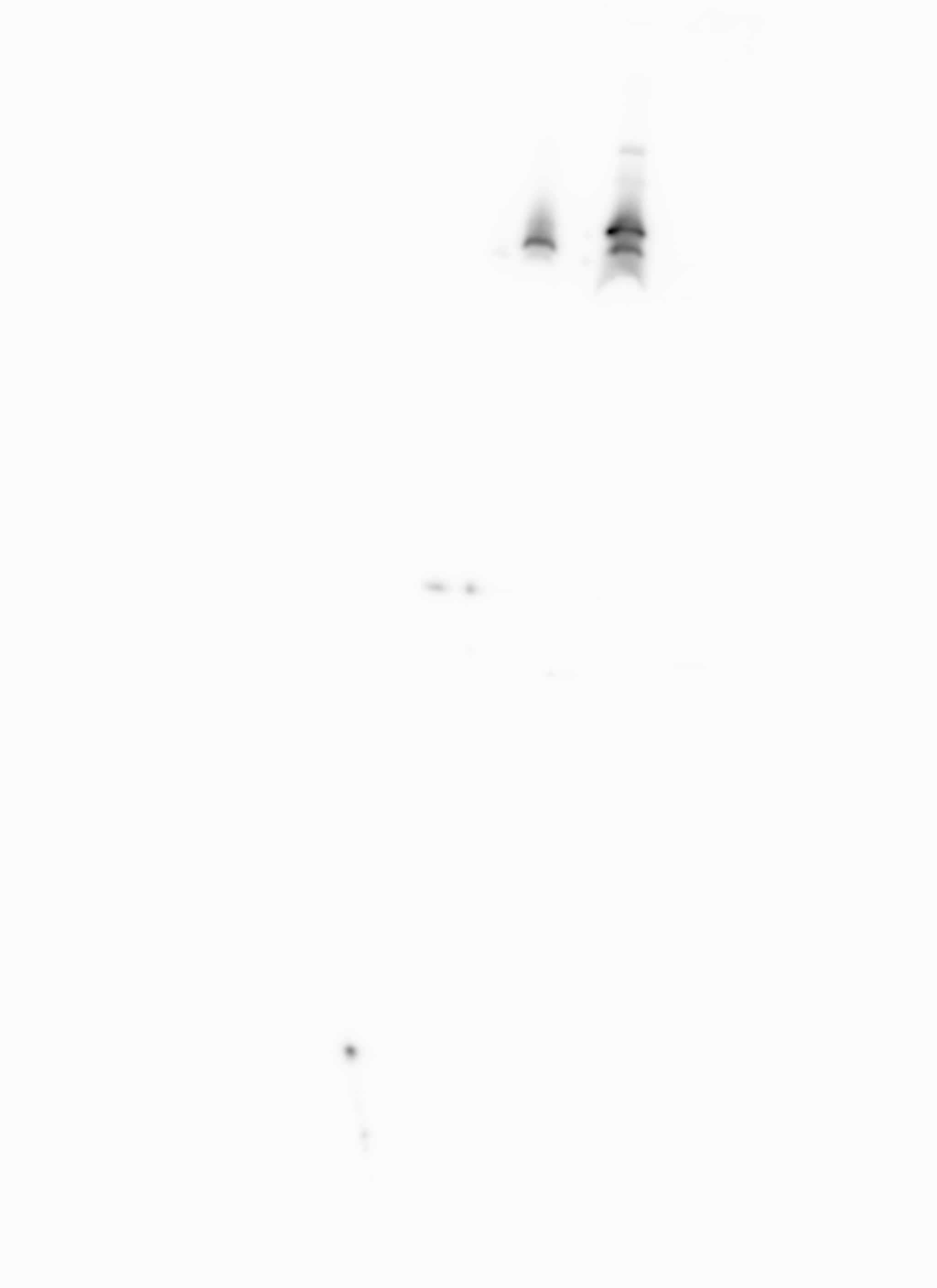

Supplement: Supplementary file 8 — Source Data [file 41467_2022_28503_MOESM8_ESM.zip › FigS2c_MTCO1_SDHB.tif]

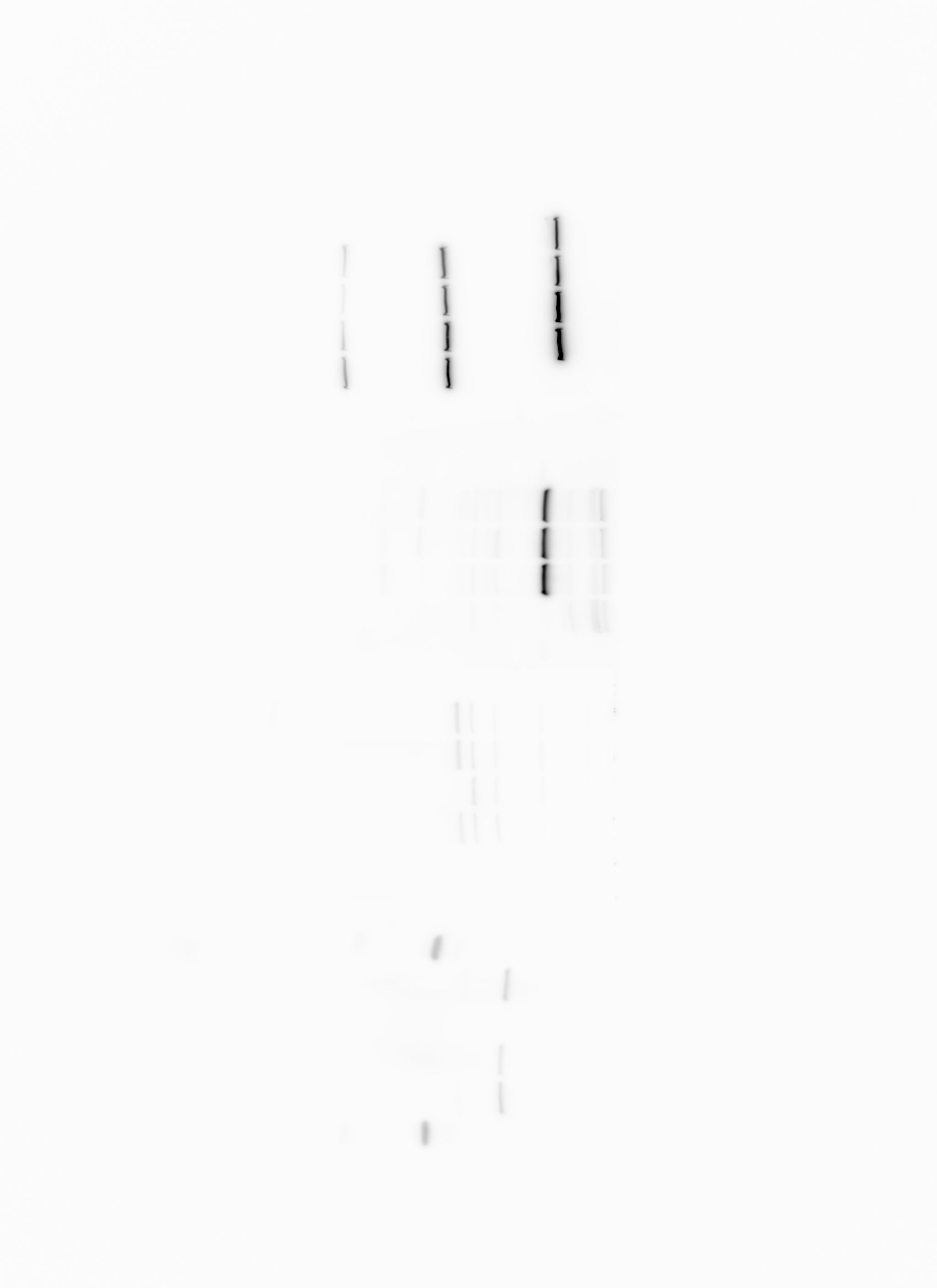

Supplement: Supplementary file 8 — Source Data [file 41467_2022_28503_MOESM8_ESM.zip › FigS1_MRM1_MRM2_MRM3_Vinculin.tif]

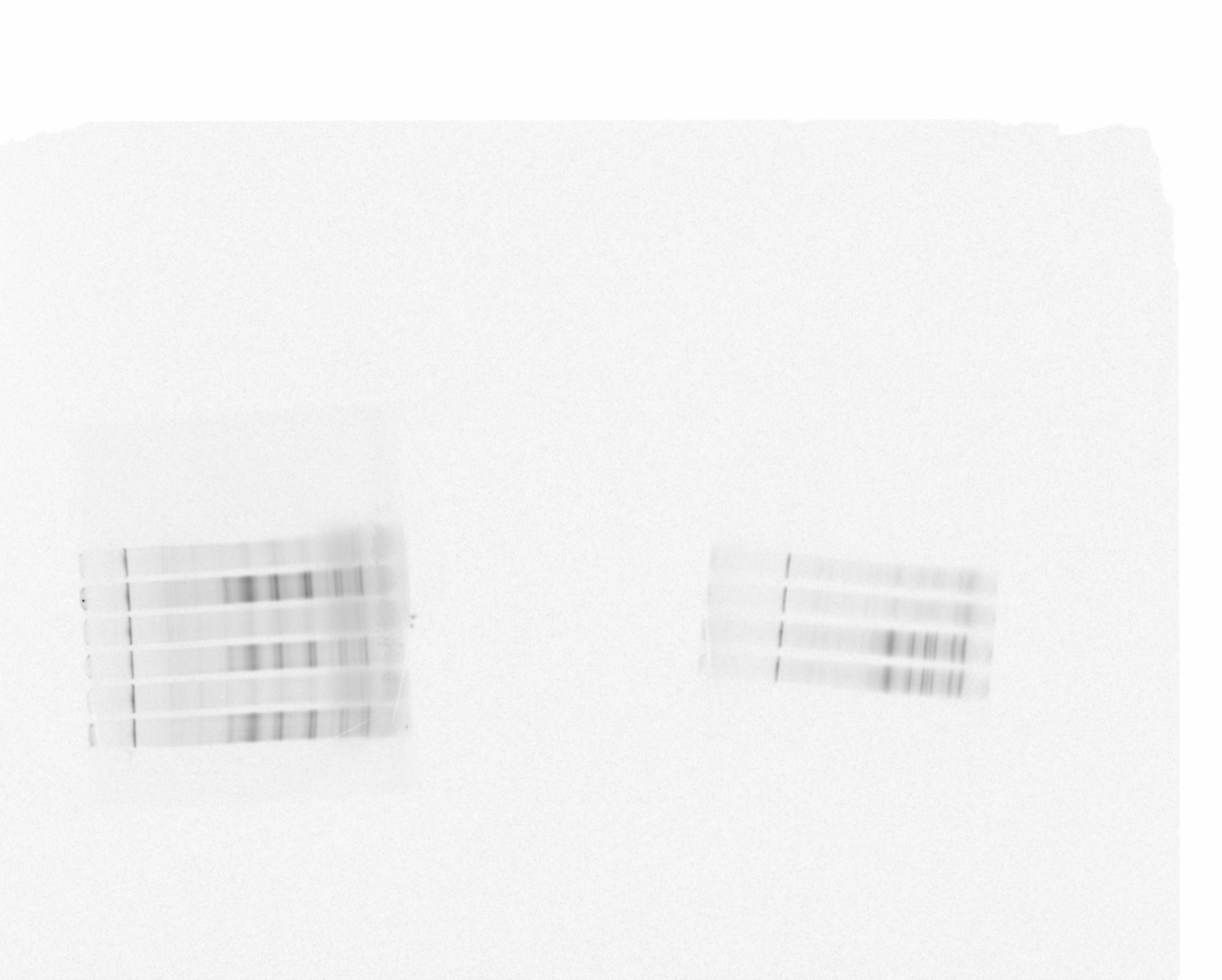

Supplement: Supplementary file 8 — Source Data [file 41467_2022_28503_MOESM8_ESM.zip › Fig3b_Autoradiography.gel]

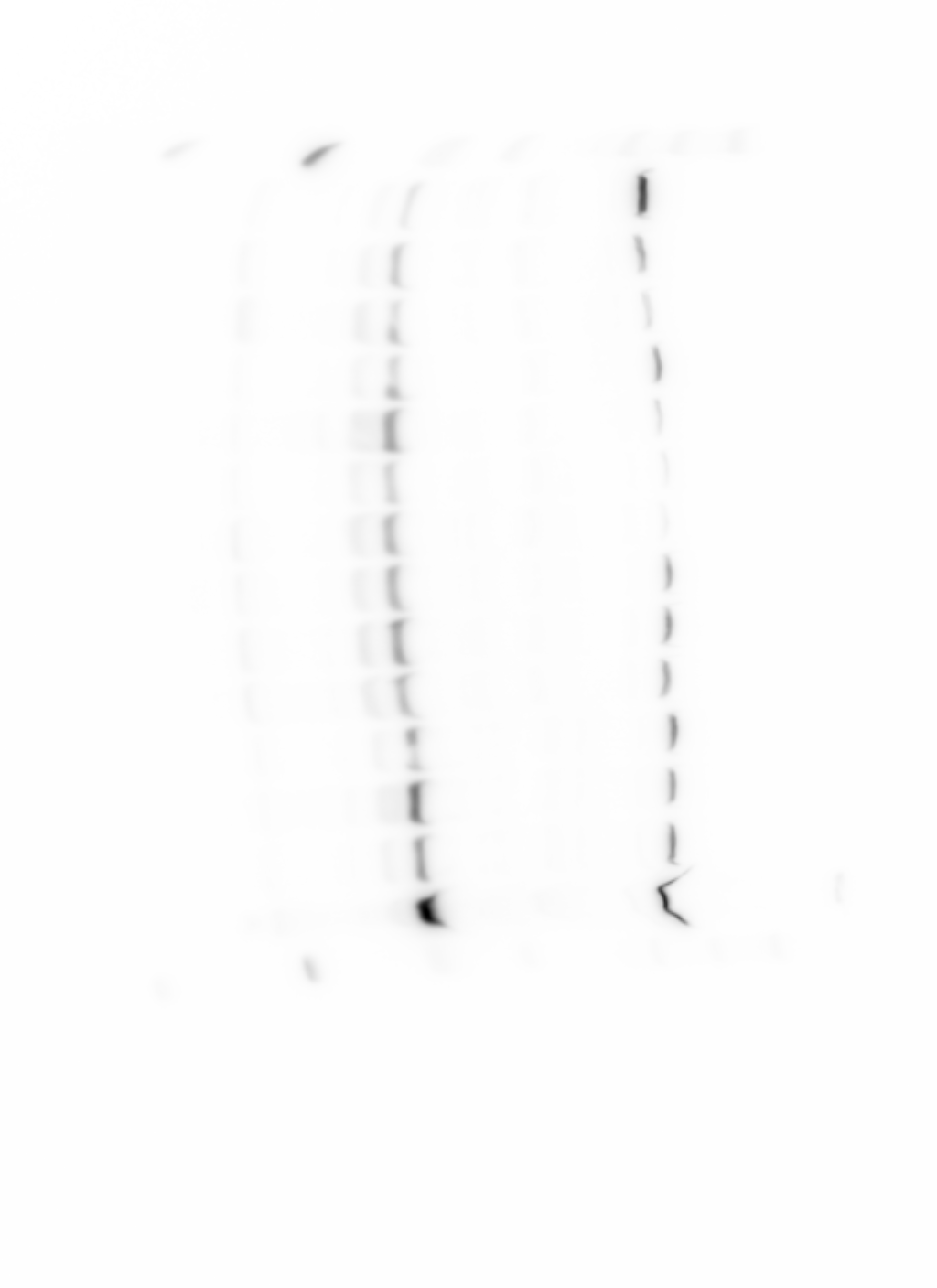

Supplement: Supplementary file 8 — Source Data [file 41467_2022_28503_MOESM8_ESM.zip › FigS11_GTPBP7.tif]

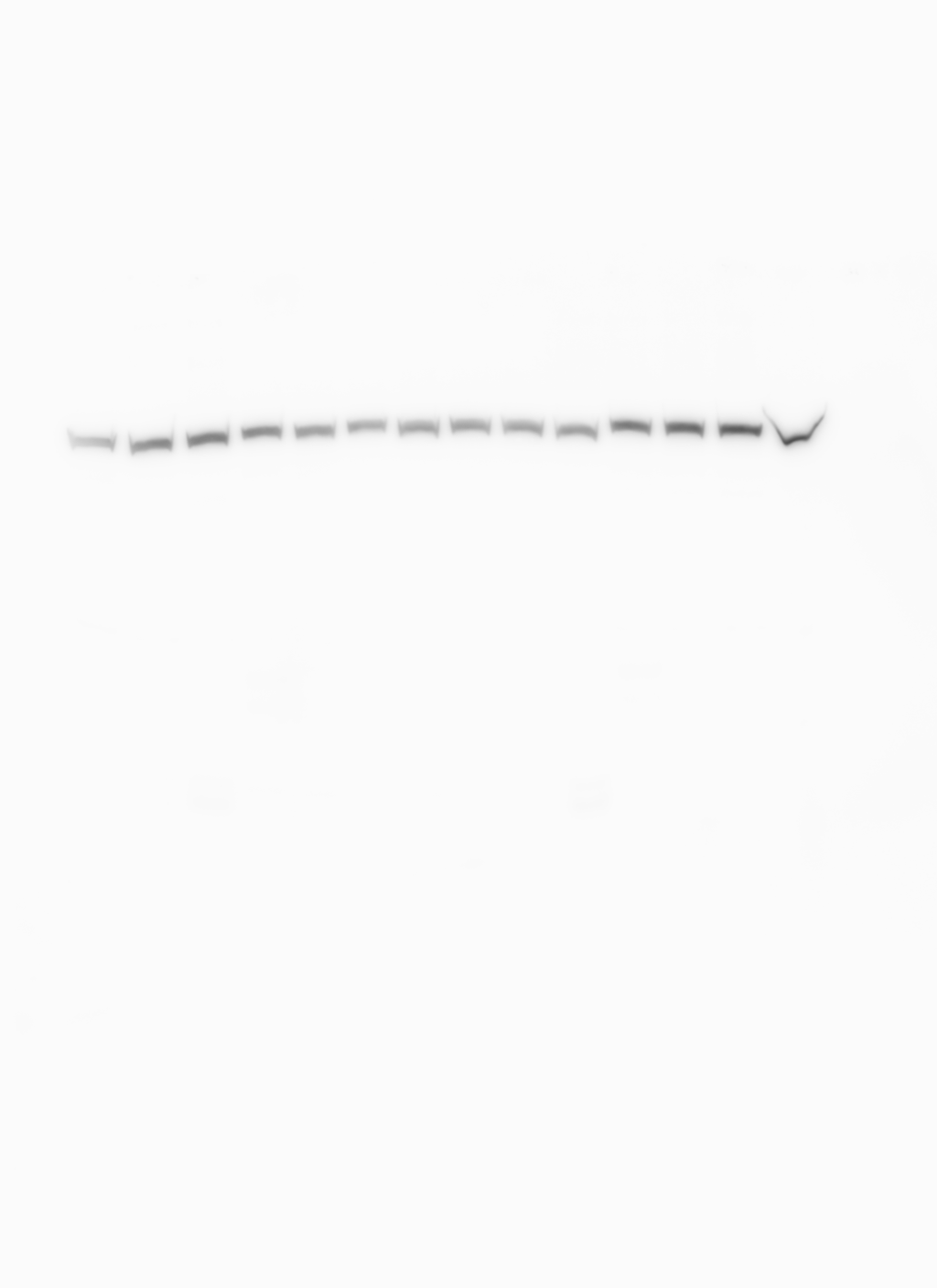

Supplement: Supplementary file 8 — Source Data [file 41467_2022_28503_MOESM8_ESM.zip › FigS11_Vinculin.tif]

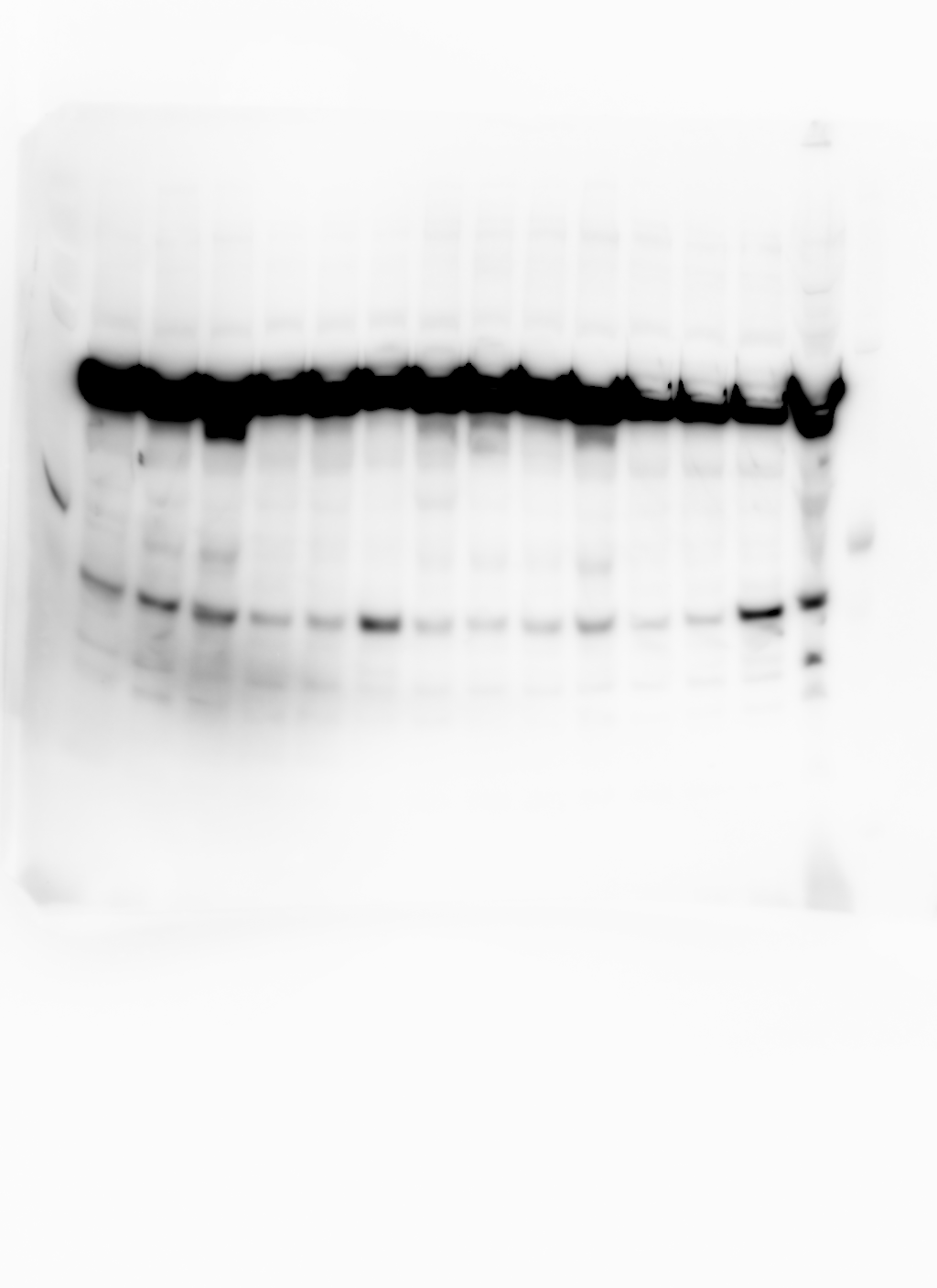

Supplement: Supplementary file 8 — Source Data [file 41467_2022_28503_MOESM8_ESM.zip › FigS11_GTPBP8.tif]

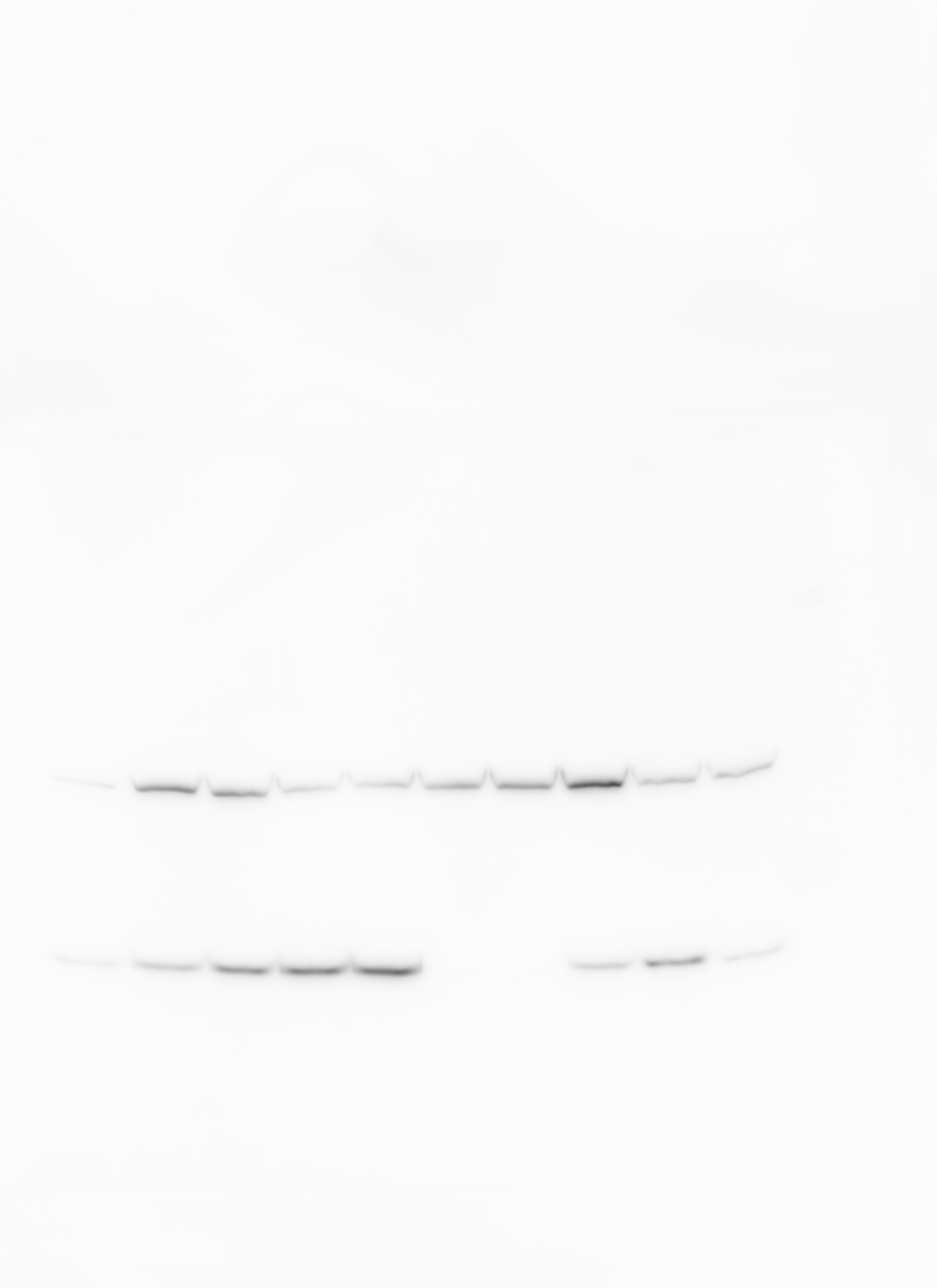

Supplement: Supplementary file 8 — Source Data [file 41467_2022_28503_MOESM8_ESM.zip › Fig6c_MTCO2.tif]

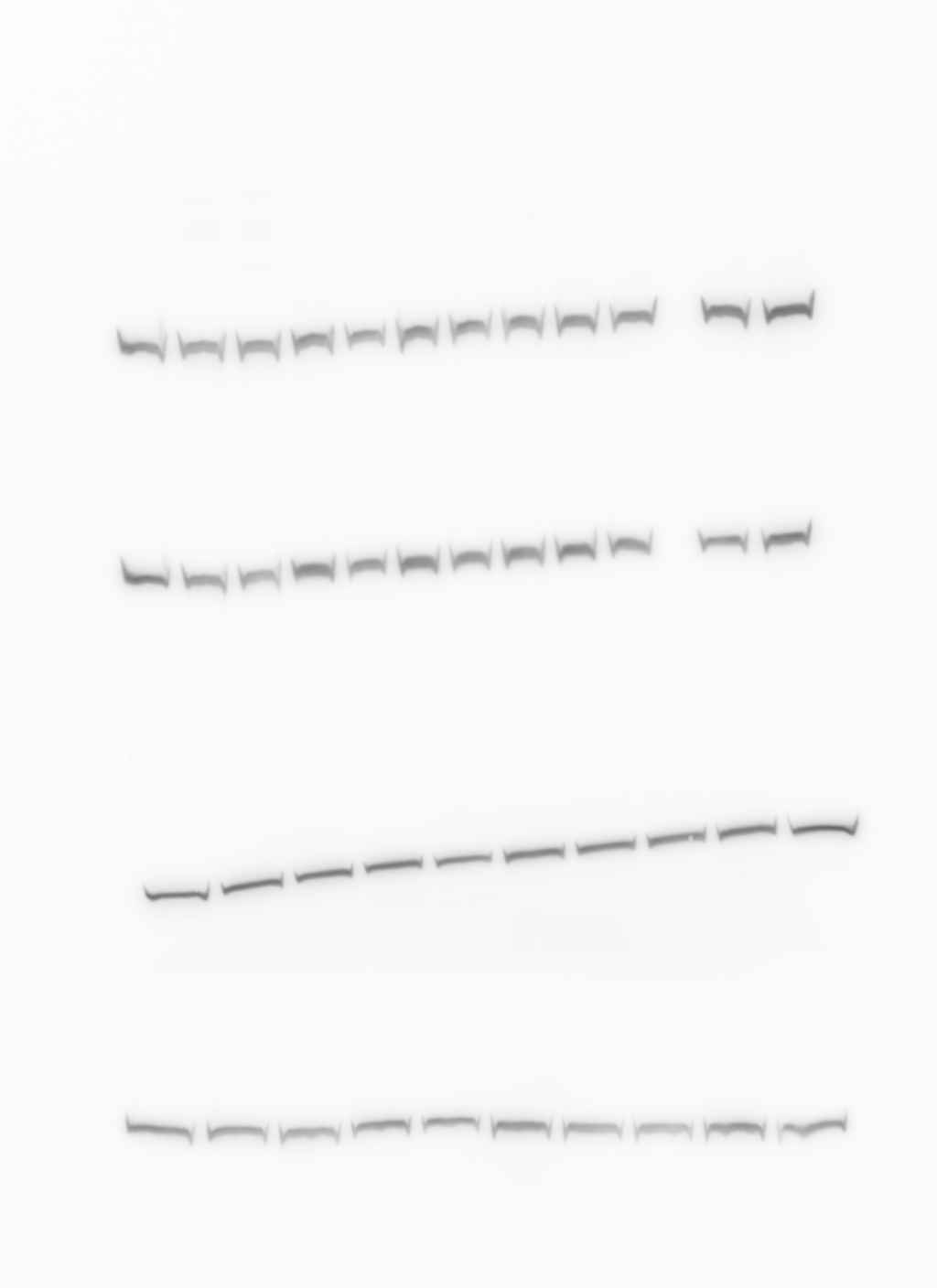

Supplement: Supplementary file 8 — Source Data [file 41467_2022_28503_MOESM8_ESM.zip › Fig6c_Vinculin.tif]

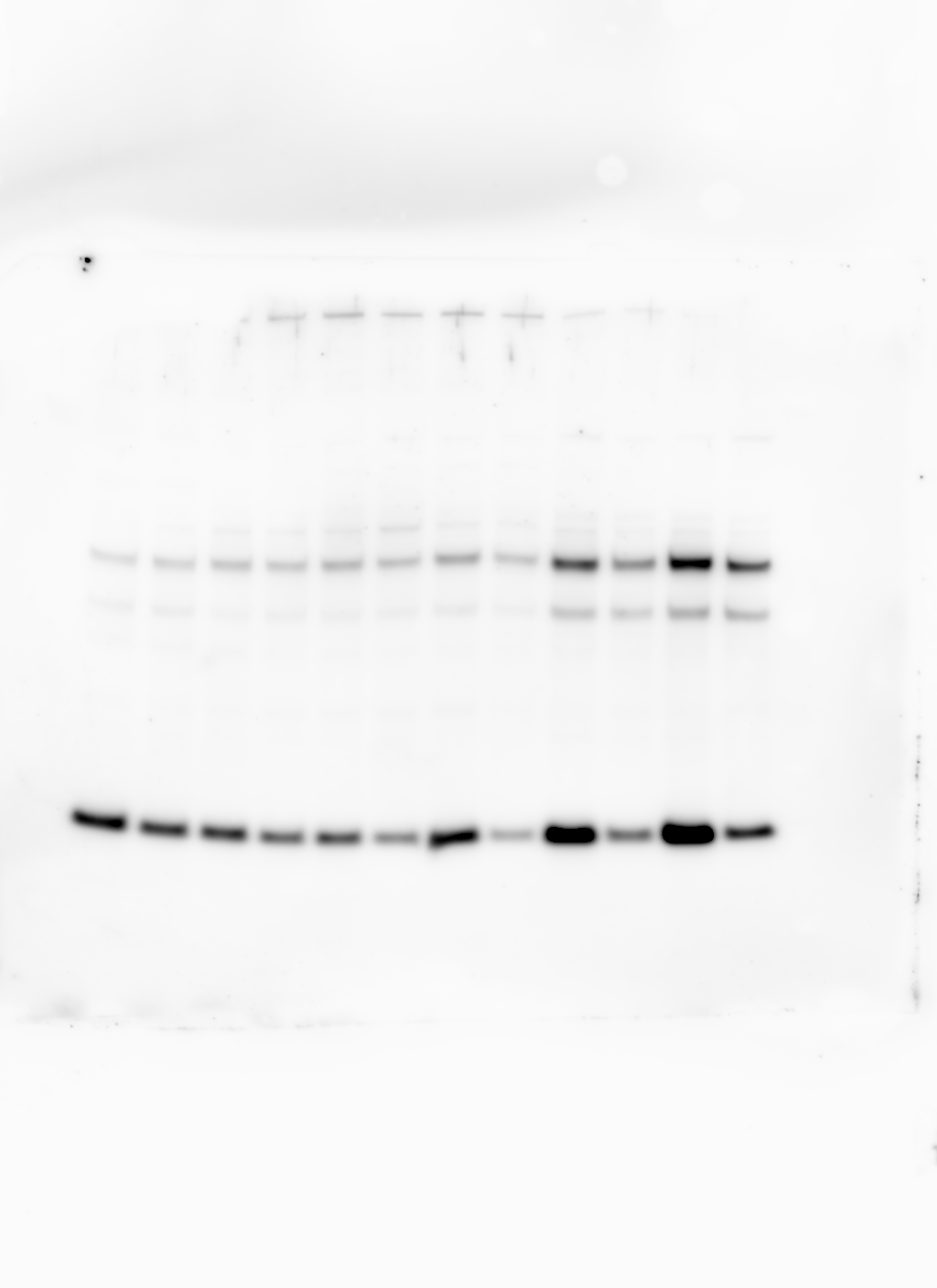

Supplement: Supplementary file 8 — Source Data [file 41467_2022_28503_MOESM8_ESM.zip › Fig7c_mtND1.tif]

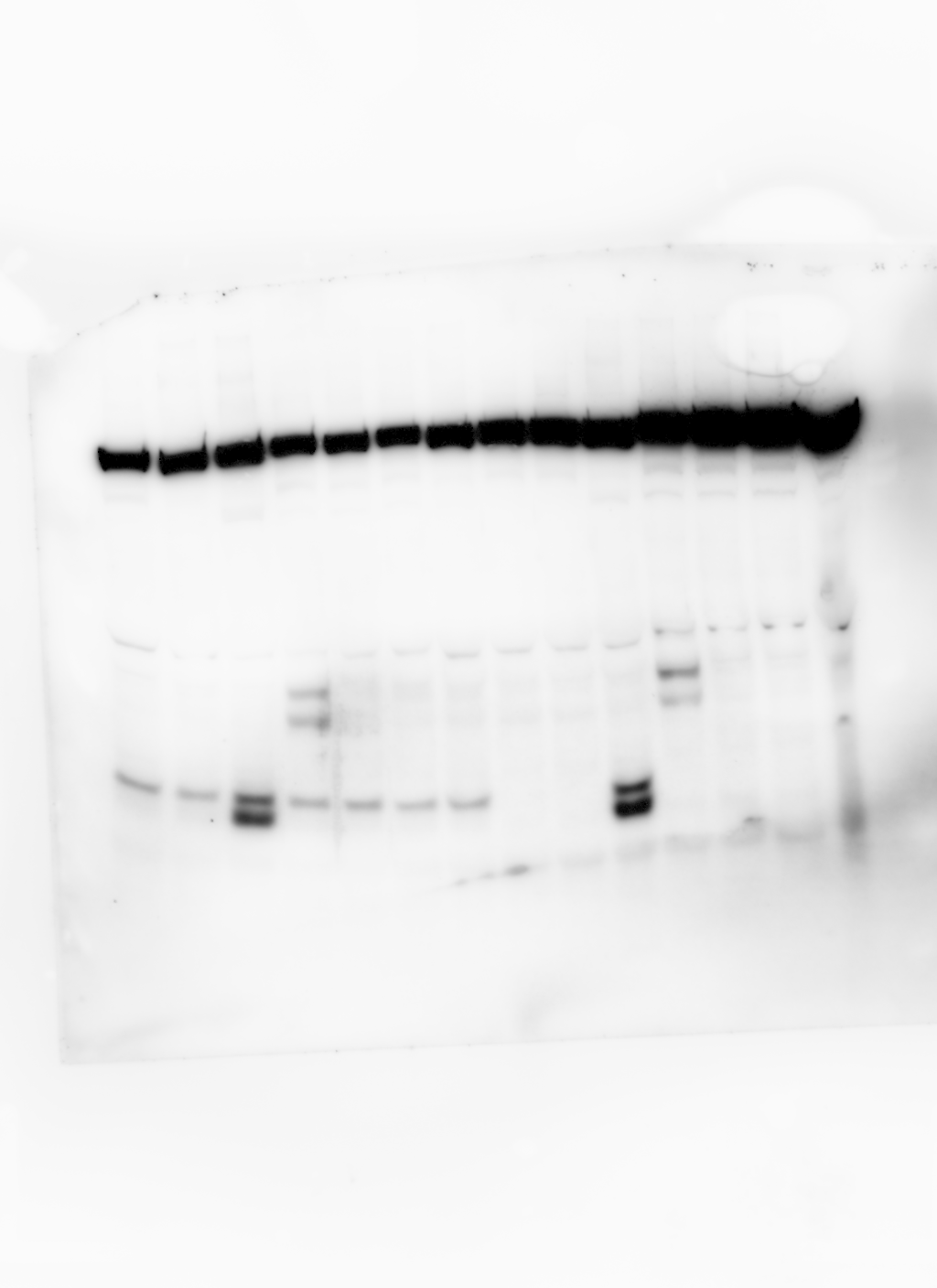

Supplement: Supplementary file 8 — Source Data [file 41467_2022_28503_MOESM8_ESM.zip › FigS11_GTPBP5_MRM2.tif]

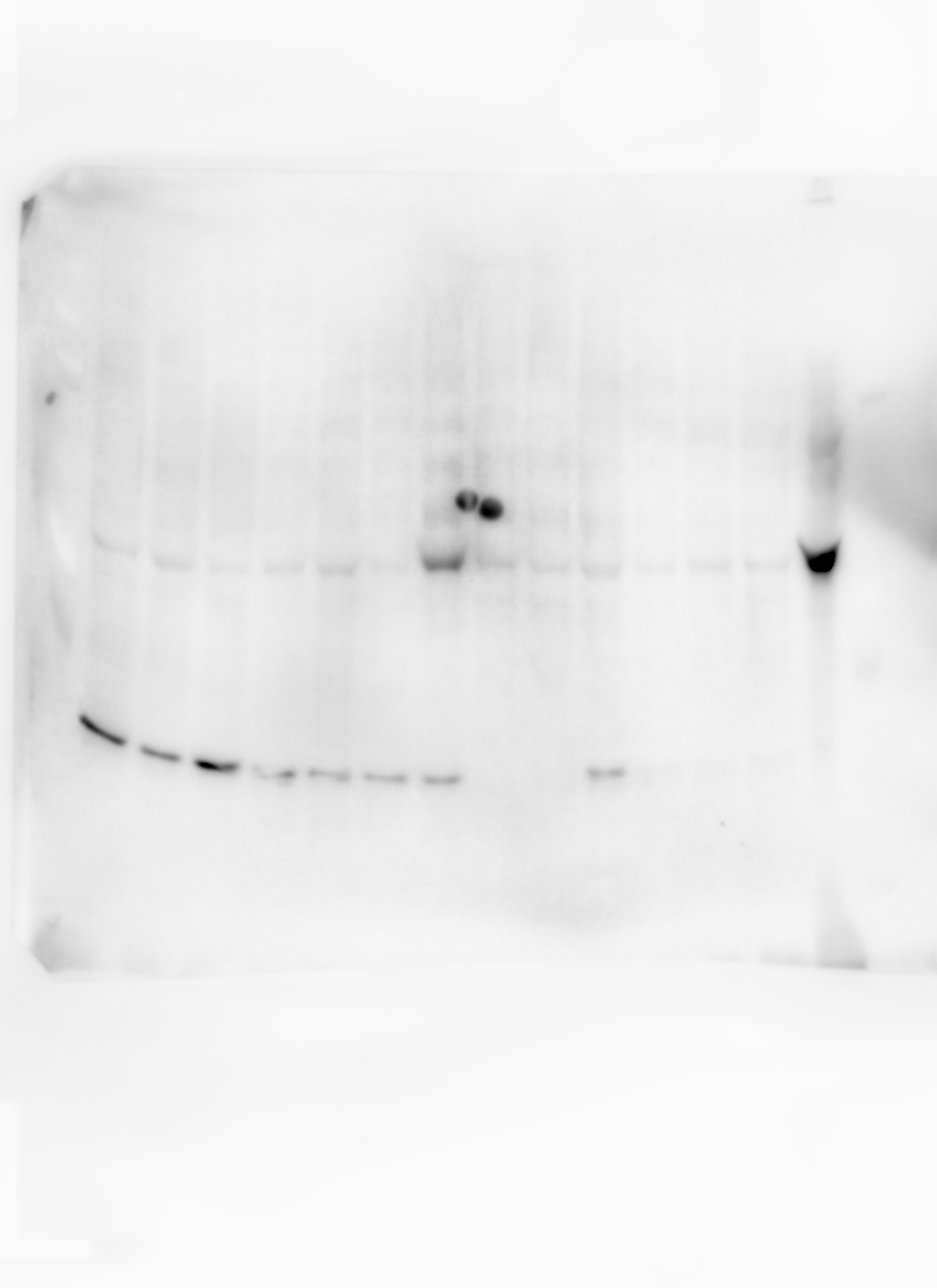

Supplement: Supplementary file 8 — Source Data [file 41467_2022_28503_MOESM8_ESM.zip › FigS11_GTPBP10_MTCO2.tif]
